# Integrated multiomics analysis reveals the molecular mechanism of light intensity-enhanced healing in cotyledon-less splice grafted watermelon

**DOI:** 10.1093/hr/uhaf293

**Published:** 2025-10-30

**Authors:** Yehia Abouseif, Akebaierjiang Kadeer, Haishun Cao, Muhammad Mohsin Kaleem, Michitaka Notaguchi, Qifan Xie, Jun Qing, Zhilong Bie, Yuan Huang

**Affiliations:** National Key Laboratory for Germplasm Innovation and Utilization of Horticultural Crops, College of Horticulture and Forestry Sciences, Huazhong Agricultural University, Wuhan 430070, Hubei Province, China; Horticulture Research Institute, Agricultural Research Center, Giza 12119, Egypt; National Key Laboratory for Germplasm Innovation and Utilization of Horticultural Crops, College of Horticulture and Forestry Sciences, Huazhong Agricultural University, Wuhan 430070, Hubei Province, China; Institute of Facility Agriculture, Guangdong Academy of Agricultural Sciences, Guangzhou 510640, China; National Key Laboratory for Germplasm Innovation and Utilization of Horticultural Crops, College of Horticulture and Forestry Sciences, Huazhong Agricultural University, Wuhan 430070, Hubei Province, China; National Key Laboratory for Germplasm Innovation and Utilization of Horticultural Crops, College of Horticulture and Forestry Sciences, Huazhong Agricultural University, Wuhan 430070, Hubei Province, China; Department of Botany, Graduate School of Science, Kyoto University, Kitashirakawa Oiwake-cho, Sakyoku, Kyoto 606-8502, Japan; Guangxi Academy of Sericultural Sciences, Nanning 530007, China; Guangxi Academy of Sericultural Sciences, Nanning 530007, China; National Key Laboratory for Germplasm Innovation and Utilization of Horticultural Crops, College of Horticulture and Forestry Sciences, Huazhong Agricultural University, Wuhan 430070, Hubei Province, China; National Key Laboratory for Germplasm Innovation and Utilization of Horticultural Crops, College of Horticulture and Forestry Sciences, Huazhong Agricultural University, Wuhan 430070, Hubei Province, China

## Abstract

Grafting in watermelon using traditional methods often causes rootstock regrowth, increasing labor demand and production costs. Although cotyledon-less splice grafting eliminates regrowth by excising meristem tissue, its success rate has consistently been lower. Here, we developed a novel cotyledon-less splice grafting methodology that achieved high survival rates by modulating pre-grafting light intensities from 100 to 300 μmol·m^−2^·s^−1^ for scion and rootstock, generating four experimental groups: high-light intensity scion/high-light intensity rootstock (HS/HR), high-light intensity scion/low-light intensity rootstock (HS/LR), low-light intensity scion/high-light intensity rootstock (LS/HR), and low-light intensity scion/low-light intensity rootstock (LS/LR). The results demonstrated that HS/HR and LS/HR exhibited the highest survival rates, nearly 98%, and displayed high seedling quality, markedly enhanced graft-union adhesion, and accelerated vascular reconnection. Pretreatment of high light intensity increased starch accumulation in rootstock hypocotyls, enhancing tolerance to carbon starvation after grafting especially in the cotyledon-less grafts. Metabolomic analysis identified elevated levels of key metabolites, including auxins, cytokinins, D-galactose, galactinol, starch, cinnamic acid, M-coumaric acid, and vanilloloside. Transcriptomic profiling revealed significant enrichment of plant hormone signal, starch and sucrose metabolism, and phenylpropanoid biosynthesis pathways in scion and rootstock tissues underpinning hormonal regulation, carbohydrate metabolism, and lignin biosynthesis under high-light conditions. WGCNA identified key co-expression modules associated with graft healing traits and key metabolites. Furthermore, graft healing related genes (*PXY*, *NAC086*, *CALS7*, and *TMO6*) were upregulated. In conclusion, our findings underscore the critical role of light intensity in orchestrating transcriptional and metabolic networks to optimize graft healing, providing a physiological and molecular foundation for improving cotyledon-less grafting efficiency.

## Introduction

Grafting has emerged as a widely adopted technique in watermelon production, serving as a promising strategy for managing biotic and abiotic stresses [1–3], and enhancing fruit yield and quality [4, 5]. The predominant commercial watermelon grafting methods, hole insertion and one cotyledon, often retain rootstock bud meristems, causing shoot regrowth rates of up to 73% in some cases [6]. Rootstock shoot regrowth competes with the scion for resources leading to graft failure, reducing yields, and hindering grafting adoption in commercial systems [7]. Additionally, rootstock shoot regrowth significantly increases the cost of grafted transplants. Grafted seedlings can be up to five times more expensive than nongrafted due to labor-intensive monitoring and shoot regrowth removal, which accounts for 48%–60% of manual grafting expenses [8, 9]. Cotyledon-less splice grafting without rootstock cotyledons eliminates shoot regrowth by removing meristem tissue below the axillary bud [10, 11]. However, this technique often results in lower survival rates and poorer seedling quality as cotyledons serve as the primary source of hormones and carbohydrates during the critical healing phase, and their absence leads to significantly lower survival rates than traditional methods that retain at least one cotyledon [8]. Thus, improving this emerging grafting technique is essential for enhancing efficiency and reducing costs.

The success of grafting depends on vascular reconnection between the scion and rootstock [12, 13]. Phytohormones are vital for tissue attachment, callus formation, and vascular bundle development, leading to graft success [14, 15]. The grafting union exhibits rapid vascular reconnection and induces differential gene expression related to auxin and cell wall remodeling [16]. Auxin promotes cell division in procambial tissues and is critical in graft union establishment [17]. In addition, phytohormone levels in the rootstock are crucial for grafting, as auxin-enriched rootstocks accelerate graft healing [18]. Cotyledons, a key auxin source, promote vascular cell proliferation during graft union formation, inhibiting graft reunion by cotyledon excision or auxin inhibitors [19]. Rootstock cotyledons regulate callus formation through *ClPIN1a*-mediated endogenous auxin release, facilitating graft union development [20]. Furthermore, cytokinins are crucial for graft formation, as cytokinin-induced *WIND1* promotes callus formation and vascular reconnection [21]. Cytokinin levels peak below the graft junction 12 h after grafting, promoting xylem and phloem patterning in the tomato graft union [22]. The presence of sugars and proper energy levels at the graft junction is essential for callus production, vascular reconnection, and overall graft success [23]. Increased soluble sugar levels in rootstock tissue enhance grafting survival [8, 24]. Removing rootstock cotyledons reduces carbohydrate availability, lowering watermelon splice-graft survival [7]. Additionally, rootstock cotyledons, particularly their starch content, serve as a crucial buffer in the growth regulation of cucumber-pumpkin heterografts [25].

On the other hand, plant factories with artificial lighting (PFALs) present a promising solution, enabling year-round production of high-quality, uniform scions and rootstocks through precise control of growth conditions, reduced labor, and shorter production cycles [26]. Light intensity, particularly photosynthetic photon flux (PPF), is the most critical environmental factor in PFALs for optimal seedling growth and vitality [27]. Light regulate genes for phytohormones biosynthesis, signaling, and meristem development, impacting both callus formation and organ regeneration [28]. Light signaling influences the synthesis and transport of auxins and cytokinins, which have been recently shown to be essential for cell division, and vascular differentiation during grafting healing [29, 30]. The coordinated action of auxin and cytokinin triggered by light signals supports both wound-responsive callus formation and the re-establishment of vascular connectivity [14]. Phytochromes (*PHY*) and cryptochromes (*CRY*), two important light receptors, govern hormonal crosstalk at the graft site, influencing callus development and vascular reconnection [31]. Grafting studies in tomato shows that mixed red–blue LEDs light spectra after grafting accelerate graft union development by enhancing auxin-related gene expression (such as *AUX1*, *ARF30*, and *LAX3*), which improve the vascular differentiation [32]. Similarly, increased light intensity promotes root growth in seedlings by enhancing the expression of genes associated with phytohormone signaling pathways, and cell wall remodeling particularly through the interaction of *HY5* and *RHD6* [33]. High light intensities enhance carbohydrate accumulation and upregulate genes related to sucrose and starch synthesis in seedlings [34]. Moreover, high light intensity drives development and metabolism, including metabolite accumulation, in tomato seedlings [35].

Despite evidence that light intensity affects seedling vigor, its molecular role in grafting healing remains poorly characterized. To our knowledge, no study has integrated transcriptomic and metabolomic analyses to explore how pre-grafting light intensity influences healing in cotyledon-less grafting seedlings. Here, we hypothesized that pre-grafting light intensity in PFALs modulates hormone signaling, carbohydrate metabolism and phenylpropanoid-mediated lignin deposition to enhance vascular reconnection and improve survival rates in cotyledon-less watermelon grafts. We evaluated the impact of scion and rootstock exposure to 100 and 300 μmol·m^−2^·s^−1^ LED light under a 14/10-h light/dark cycle across four treatments. Survival rate, seedling quality after grafting, phloem and xylem reconnection, and biochemical indicators, including hormones and sugars during graft healing, were assessed. Furthermore, transcriptome sequencing and non-targeted metabolomics were utilized to construct a core regulatory network, revealing key metabolic pathways underlying the effects of high light intensity on survival and grafting success. This study provides new insights into optimizing PFAL light strategies to suppress rootstock regrowth and enhance the efficiency of cotyledon-less splice grafted watermelon transplants, strengthening the foundation for grafted watermelon production.

## Results

### High light intensity for rootstock enhances grafting success and root regeneration in cotyledon-less watermelon seedlings

To assess the impact of pre-grafting artificial light intensities on grafting success and seedling performance, we quantified survival rates, biomass accumulation, and root regeneration capacity in cotyledon-less watermelon–pumpkin grafts at 14 days after grafting (DAG). The results showed that using high light intensity (300 μmol·m^−2^·s^−1^) for pumpkin rootstock cultivation significantly enhanced the survival rate of splice-grafted watermelon seedlings without rootstock cotyledons.

**Figure 1:**
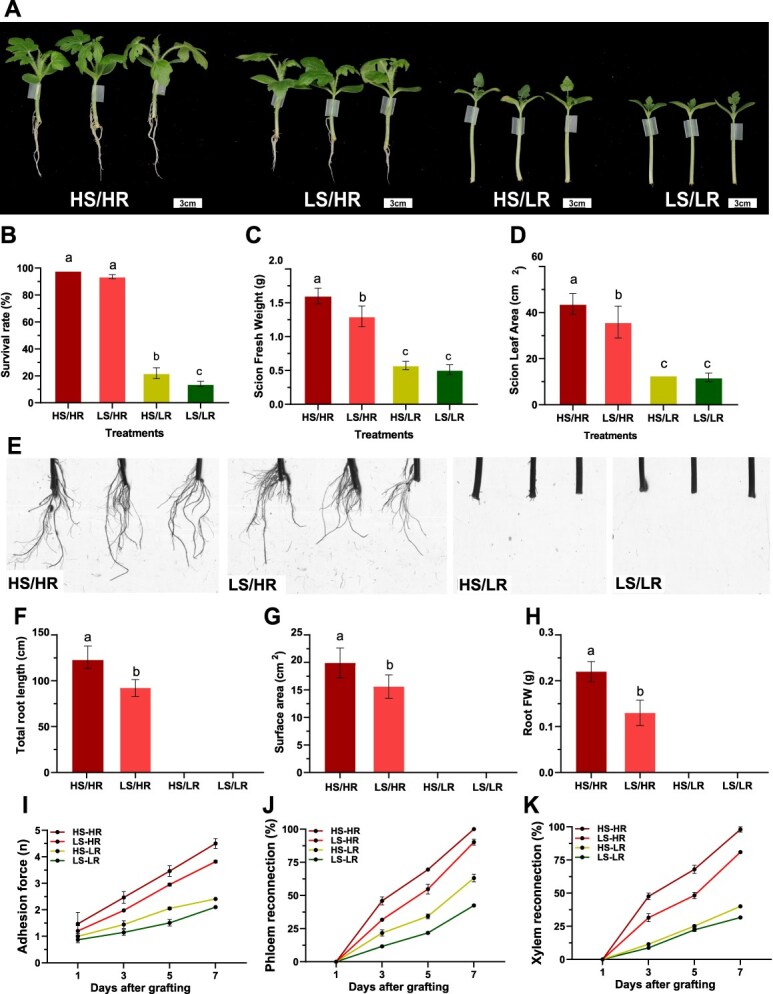
High light intensity for rootstock enhances grafting success and root regeneration in cotyledon-less grafted watermelon seedlings. A, Growth of grafted seedlings at 14 days after grafting (DAG) under four light treatments: HS/HR (high light scion/high light rootstock), LS/HR (low light scion/high light rootstock), HS/LR (high light scion/low light rootstock), and LS/LR (low light scion/low light rootstock). Scale bar = 3 cm. B, Survival rate (%) at 14 DAG. C, Scion fresh weight (g). D, Scion leaf area (cm^2^). E, Root morphology at 14 DAG. F, Total root length (cm). G, Root surface area (cm^2^). H, Root fresh weight (g). I, Adhesion force (N) of graft unions at 1, 3, 5, and 7 DAG. J, Phloem reconnection (%) during healing. K, Xylem reconnection (%) during grafting healing. Data are presented as mean ± SE (*n* = 3). Different letters above bars indicate significant differences among treatments (*P* < 0.05, one-way ANOVA).

In contrast, scion light intensity had no measurable impact (Fig. 1A). HS/HR and LS/HR treatments achieved nearly 98% survival at 14 DAG. In contrast, HS/LR and LS/LR dropped below 20% (Fig. 1B). These findings indicate that exposing rootstock seedlings to high light intensity before grafting, rather than scion seedlings, can improve grafting success when both cotyledons are removed from the rootstock.

Additionally, high light intensity prior to grafting significantly enhanced grafted seedling growth and quality (Fig. 1A). The HS/HR treatment exhibited the most favorable outcomes, with increased leaf area, leaf count, scion length, diameter, fresh weight, and overall biomass accumulation (Fig. 1C and D and Fig. S1). The LS/HR treatment followed this, whereas the HS/LR and LS/LR treatments showed minimal growth from the time of grafting.

Root morphology analysis revealed that rootstock seedlings exposed to high light intensity pre-grafting exhibited improved root regeneration compared to those under low light conditions (Fig. 1E). At 14 DAG, the HS/HR treatment exhibited significantly greater total root length, surface area, root volume, and fresh root weight compared to LS/HR. In contrast, HS/LR and LS/LR treatments showed minimal root development, with root emergence nearly absent (Figs 1F–H and S1).

To evaluate the role of light intensity in graft healing, we quantified adhesion force and vascular reconnection at the graft junction throughout the healing process. High-light rootstock combinations (HS/HR and LS/HR) exhibited a steady increase in adhesion force over time, whereas low-light combinations (HS/LR and LS/LR) showed only a transient initial increase, which then remained consistently low (Fig. 1I).

Similarly, phloem reconnection was significantly enhanced in the HS/HR and LS/HR treatments, reaching approximately 60% and 50% by 5 DAG and increasing to 90% and 80% by 7 DAG, respectively (Fig. 1J). In contrast, the HS/LR and LS/LR treatments exhibited delayed and much lower reconnection rates, with only 20% of seedlings showing phloem reconnection at 5 DAG, increasing modestly to 30% by 7 DAG.

Xylem reconnection followed a similar trend, with high-light rootstock treatments (HS/HR and LS/HR) achieving near-complete restoration by 7 DAG, while low-light combinations (HS/LR and LS/LR) remained severely impaired (Fig. 1K).

### Differential metabolic profiles of the grafted union in response to pre-grafting light intensities

**Figure 2:**
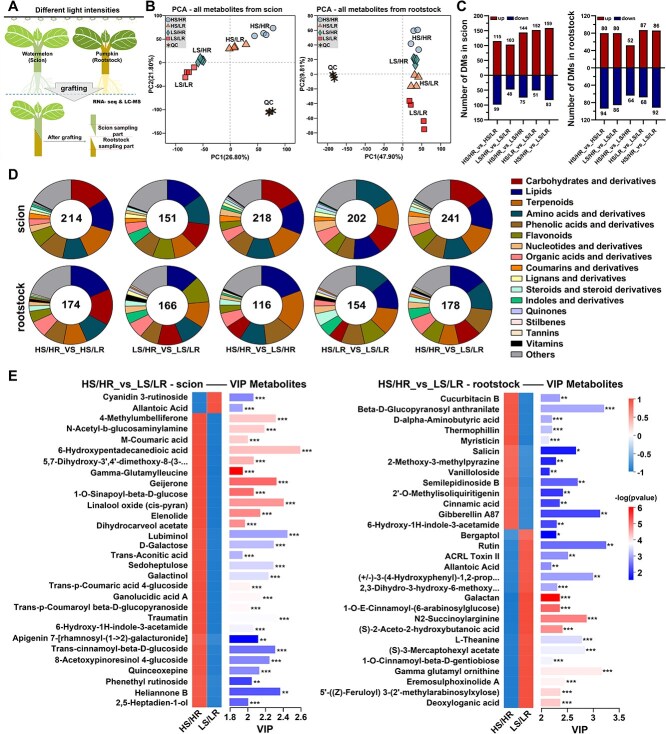
Differential metabolic profiles of the grafted union in response to pre-grafting light intensities. A, Experimental setup and sampling scheme. B, Principal component analysis (PCA) of all detected metabolites from scion and rootstock, showing clear separation among treatments. C, Number of differentially accumulated metabolites (DAMs) in scion and rootstock across comparisons, indicating upregulated and downregulated metabolites. D, Functional classification of DAMs in scion and rootstock grouped into major categories, including carbohydrates, lipids, amino acids, phenolic acids, flavonoids, and terpenoids. E, Variable importance in projection (VIP) analysis of scion and rootstock metabolites in HS/HR vs. LS/LR comparison. Heatmaps show relative metabolite abundance, with VIP scores on the x-axis. Data represent the mean of four independent biological replicates per treatment. Asterisks indicate statistical significance: ^*^P < 0.05, ^**^P < 0.01, ^***^P < 0.001.

LC–MS was employed to identify metabolites associated with graft union formation and investigate metabolic dynamics in scion and rootstock tissues in response to light intensity treatments in grafted watermelon seedlings. The experimental setup and sampling followed the method described in Fig. 2A. Metabolite profiling identified 765 metabolites, including carbohydrates, amino acids, organic acids, nucleotides and their derivatives, lipids, flavonoids, and terpenoids (Table S1). Differentially accumulated metabolites (DAMs) were analyzed using principal component analysis (PCA) and showed significant differences between scion and rootstock samples across different treatments, while minimal variation was observed among biological replicates, (Fig. 2B).

As shown in Fig. 2C, the number of DAMs identified in scion and rootstock samples varied across comparisons, with the distribution of upregulated and downregulated DAMs. The hierarchical clustering heatmap of key DAMs further highlighted distinct patterns in their regulation between scion and rootstock. Metabolomic profiling revealed the accumulation of hormone-related metabolites (auxin and cytokinin derivatives), carbohydrate metabolites, and lignin precursors (Fig. S2). Their common detection across clusters suggests an integrated role under high light, where hormone signaling works in concert with carbohydrates and lignin metabolic that supply energy and structural components to support tissue regeneration and vascular reconnection at the graft union. DAMs were classified into 17 metabolic categories (Fig. 2D), revealing unique metabolite abundance patterns between scion and rootstock samples. Notably, carbohydrates, lipids, terpenoids, and amino acids exhibited differential accumulation across treatments.

KEGG enrichment analysis for all comparisons in the scion identified significant enrichment in pathways related to nucleotide metabolism, ABC transporters, phenylpropanoid biosynthesis, and carbohydrate metabolism, including galactose metabolism, pentose metabolism, TCA cycle, and ascorbate and aldarate metabolism. KEGG enrichment analysis for all comparisons in the rootstock revealed enrichment in pathways associated with plant hormone signal transduction, amino acids that involved in hormones biosynthesis (e.g. tryptophan metabolism and zeatin biosynthesis), phenylpropanoid metabolism (e.g. phenylalanine metabolism and flavonoid metabolism) and amino acid metabolism (e.g. alanine, aspartate, leucine, glutamate, glutathione and tyrosine) (Fig. S3; Table S2).

Using an OPLS-DA model with a threshold of fold change >1 and VIP > 1 for the HS/HR vs. LS/LR comparison, which represents the greatest contrast in grafting success, the top 30 metabolites were identified (Fig. 2E), and supplemented in Table S3. In the scion, metabolites related to carbohydrate metabolism (D-galactose, galactinol, and sedoheptulose), hormones derivatives (6-hydroxy-1H-indole-3-acetamide), and the phenylpropanoid pathway (m-coumaric acid and trans-*p*-coumaric acid 4-glucoside) were more abundant in HS than LS (Fig. 2E). In the rootstock, metabolites related to hormones derivatives (6-hydroxy-1H-indole-3-acetamide and gibberellin A87) and phenylpropanoid pathway (cinnamic acid, vanilloloside, and salicin) which exhibited higher VIP values in HR rootstock than in LR (Fig. 2E).

This accumulation of metabolites resulted from enhanced metabolic activation in response to increased light intensity. High light intensity promotes photosynthetic activity and carbon assimilation, thereby elevating sugar precursors such as D-galactose and galactinol, which serve as energy and carbon skeletons sources for cell division, cell wall biosynthesis and callus proliferation. Meanwhile, indole-based hormone derivatives indicate auxin-related signaling activity, which is known to regulate cell division and vascular reconnection. Phenylpropanoid intermediates compounds contribute to lignin biosynthesis and oxidative stress management, reinforcing cell walls and lignification processes crucial for graft union stabilization.

### Transcriptional dynamics of graft union in response to varying light intensities

RNA-seq was employed to investigate the molecular mechanisms underlying the early grafting response between the scion and rootstock under different light-intensity conditions. The sequencing of 24 samples including three replicates, yielded 163.57 Gb of clean data, with an average 96.16% of bases scoring Q30 (Table S3). Clean reads were aligned separately to the *Citrullus lanatus* (97103) v2 reference genome and the *Cucurbita moschata* (Rifu) reference genome for scion and rootstock, respectively. Unique alignment rates ranged from 84.82% to 97.93% across the samples. A total of 28 703 and 19 008 uniquely expressed genes were identified in the watermelon scion and pumpkin rootstock samples, respectively (Table S4).

**Figure 3:**
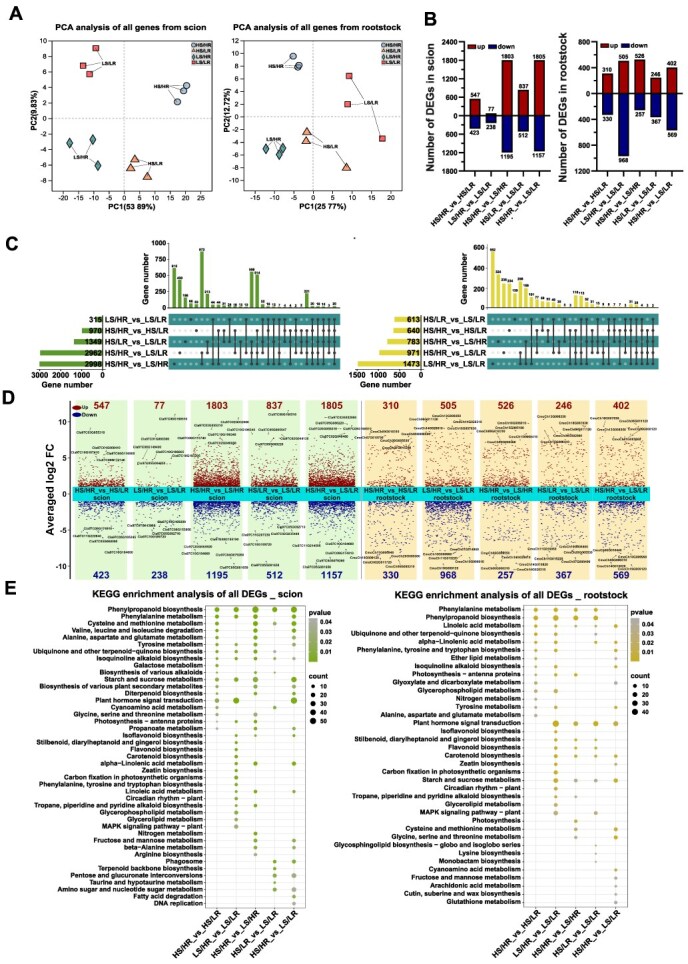
Transcriptional dynamics of graft union in response to varying light intensities. A, Principal component analysis (PCA) of gene expression profiles in scion and rootstock under different light treatments. B, Numbers of differentially expressed genes (DEGs; up- and down-regulated) in scion and rootstock across comparisons. C, UpSet plots showing overlaps of DEGs among treatments in scion and rootstock. D, Volcano plots illustrating up- and down-regulated DEGs in representative comparisons. E, KEGG pathway enrichment analysis of DEGs in scion and rootstock, highlighting significantly enriched biological processes and metabolic pathways. Data are shown as mean ± SE (*n* = 3).

PCA revealed clear distinctions in transcriptome profiles between scion and rootstock samples across different treatments, with minimal variation among the three biological replicates for each sample, confirming the dataset’s suitability for subsequent analyses (Fig. 3A). Transcriptome sequencing of watermelon scion and pumpkin rootstock identified differentially expressed genes (DEGs) across five comparison groups: HS/HR vs. HS/LR, LS/HR vs. LS/LR, HS/HR vs. LS/HR, HS/LR vs. LS/LR, and HS/HR vs. HS/HR. In watermelon scion, these comparisons yielded 970, 315, 2998, 1349, and 2962 DEGs, respectively, with varying numbers of upregulated and downregulated genes (Fig. 3B). Similarly, the pumpkin rootstock comparisons identified 640, 1473, 783, 613, and 911 DEGs, with notable differences in gene expression patterns across treatments (Fig. 3B). UpSet and volcano plots illustrated overlapping DEGs between different grafting combinations (Fig. 3C and D). UpSet plot analysis revealed unique and overlapping DEG sets in both scion and rootstock tissues among different comparisons. In the scion, 559 DEGs were shared among the comparisons HS/LR vs. LS/LR, HS/HR vs. LS/LR, and HS/HR vs. LS/HR. In the rootstock, 118 DEGs were shared among the comparisons HS/HR vs. HS/LR, LS/HR vs. LS/LR, and HS/HR vs. LS/LR. This suggests a high light-dependent effect in both scion and rootstock compared with low light. These overlapping DEG sets among multiple comparisons shared the molecular mechanisms contributing to graft healing, including energy metabolism, hormone signaling, phenylpropanoid biosynthesis, cell-wall modification, and stress regulation. The strong transcriptomic reprogramming observed under high-light conditions supports the hypothesis that pre-grafting high light improves rootstock capability for healing by accelerating processes such as callus proliferation, lignin deposition, and vascular reconnection.

KEGG enrichment analysis was performed to explore the biological functions of DEGs during graft healing. In watermelon scion, KEGG analysis identified significant enrichment in pathways related to phenylpropanoid biosynthesis, phenylalanine metabolism, and starch and sucrose metabolism across the HS/HR vs. HS/LR, LS/HR vs. LS/LR, HS/HR vs. LS/HR, and HS/HR vs. LS/LR comparison groups (Fig. 3E). Additionally, plant hormone signal transduction pathway was significantly enriched in the HS/HR vs. HS/LR, LS/HR vs. LS/LR, and HS/HR vs. LS/LR comparison groups. In pumpkin rootstock, KEGG analysis revealed significant enrichment in pathways related to plant hormone signal transduction, phenylpropanoid biosynthesis, and starch and sucrose metabolism across the LS/HR vs. LS/LR, HS/HR vs. LS/HR, HS/LR vs. LS/LR, and HS/HR vs. LS/LR comparison groups (Table S5). These pathways likely play key roles in graft union formation.

Further, Gene Ontology (GO) enrichment analysis was conducted on DEGs in both scions and rootstocksacross various comparisons (Fig. S4–S6). The top GO terms for all HS vs. LS-upregulated DEGs in watermelon scion included phenylpropanoid metabolism, cell wall biogenesis, cell wall polysaccharide metabolism, and secondary metabolic process. In pumpkin rootstock all HR vs. LR-upregulated DEGs were associated with phloem development, tissue development, phloem and xylem histogenesis, auxin signaling, hydrolase activity, cellulase activity, and antioxidant activity (Fig. S7; Table S6). The observed transcriptomic changes closely aligned with metabolomic variations identified in this study.

Light intensity pre-grafting induces tissue-specific transcriptional activation of genes related to cellular mechanisms and graft union formation. We identified several early upregulated DEGs with distinct expression patterns in the scion and rootstock. Some genes were upregulated in both tissues, while others exhibited upregulation exclusively in the scion or the rootstock. Many of the genes upregulated in response to light intensity were associated with grafting-related processes, including wound repair, cell division, and cambium and vascular development (Fig. S7B).

For example, genes related to wound healing (*LOX2* and *RAP2.6 L*), cambium formation (*RUL1*, *WOX4* and *OBP4*), cell division (*CYCLIN B 1;2*, *HISTONE H4*, and *WIND1*), cell wall modification (*GH9B3*, *EXPB3*, and *CALS7*), phloem differentiation (*CLERK* and *OPS*), and xylem development (*BFN1* and *IRX3*) were significantly induced in HS scions compared with LS scions. In the rootstock, genes related to the cambium (*WOX4* and *HCA2*), cell division (*CYCLIN B 1;2* and *HISTONE H4*), cell wall modification (*GH9B3*, *EXPB3*, and *CALS7*), provasculature (*PLL1* and *TMO6*), phloem development (*NAC086*), and xylem formation (*NAC045* and *CESA4*) exhibited higher induction in HR-grafted rootstock compared to LR rootstock (Fig. S7B). This differential regulation highlights tissue-specific transcriptional programs in the scion and rootstock that coordinate graft union formation.

Wound-stress related genes (e.g. *MLP328*, *LOX2*, and *APX*) showed transient induction in all grafted and wounded treatments, while graft-healing related genes (e.g. *CYCB1;2*, *PXY*, and *HCA2*) were more strongly upregulated in HS/HR grafts, indicating their association with successful graft union formation (Fig. S8). To confirm RNA-seq reliability, 12 randomly selected genes linked to hormonal signaling, carbohydrate metabolism and phenylpropanoid biosynthesis were analyzed via qRT-PCR using primers listed in Table S7. The expression trends closely aligned with RNA-seq results, validating the dataset’s reliability (Fig. S9).

### Hormone profiling in scion and rootstock under varying light intensities

**Figure 4:**
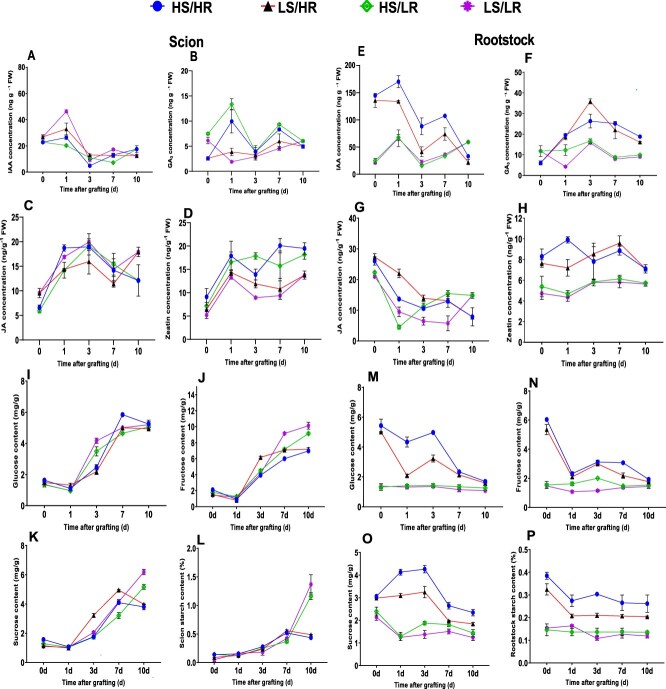
Dynamic changes in hormones and sugars in scion and rootstock during graft healing in response to pre-grafting light intensity. A–D, Hormone concentrations in scion tissues: A, Auxin (IAA); B, Gibberellic acid (GA₃); C, Jasmonic acid (JA); and D, Zeatin (ZT). E–H, Hormone concentrations in rootstock tissues: E, Auxin (IAA); F, Gibberellic acid (GA₃); G, Jasmonic acid (JA); and H, Zeatin (ZT). I–L, Sugar contents in scion tissues: I, glucose; J, fructose; K, sucrose; and L, starch. M–P, Sugar contents in rootstock tissues: M, glucose; N, fructose; O, sucrose; and P, starch. Data are shown as mean ± SE (*n* = 3).

Our findings identified several genes involved in hormone signaling pathways, prompting us to measure hormone levels in both the scion and rootstock to explore their roles in graft healing. In the scion, auxin (IAA) levels slightly increased at 1 DAG before decreasing at 3 DAG and stabilizing thereafter. Gibberellic acid (GA₃) levels peaked shortly after grafting in HS/HR and HS/LR before decreasing at 3 DAG and showing a slight increase at 7 DAG. At the onset of grafting, jasmonic acid (JA) levels were highest in HS/HR and LS/HR but rapidly declined in all treatments within the first three days. Zeatin (ZT) levels generally increased from 0 to 10 DAG, with some treatments maintaining higher concentrations (Fig. 4A–D).

The results showed distinct hormonal profiles in the rootstock, IAA concentrations were higher in HS/HR and LS/HR treatments at 0 DAG. At 1 DAG, IAA levels significantly increased across all treatments, followed by a sharp decline at 3 DAG. A slight increase was observed at 7 DAG, but by 10 DAG, IAA levels had stabilized with minimal differences among treatments. GA₃ levels slightly increased from 0 to 1 DAG in all treatments except LS/LR. This upward trend continued until 3 DAG in HS/HR and LS/HR before declining significantly across all treatments in the rootstock. By 10 DAG, the differences among treatments had narrowed as GA₃ levels continued to decrease, suggesting a reduced demand for GA₃ as graft healing progressed. JA concentrations steadily decreased over time in the rootstock, with minimal differences among treatments by 10 DAG. This pattern suggests an initial stress-related peak in JA levels, followed by a rapid decline as graft healing advanced. Zeatin levels were consistently higher in HS/HR and LS/HR than other treatments, indicating reduced cytokinin accumulation under low light conditions. While a slight increase in zeatin was observed at 1 DAG in HS/HR and LS/HR, all treatments declined at 3 DAG. However, HS/HR and LS/HR maintained higher zeatin levels than HS/LR and LS/LR at 7 DAG (Fig. 4E–H). These findings highlight the dynamic regulation of plant hormones in both the scion and rootstock during graft healing, with light intensity playing a crucial role in modulating these hormonal patterns.

### Sugar dynamics in scion and rootstock under varying light intensities

This study analyzed glucose, fructose, sucrose, and starch contents to examine their roles in graft healing. In the scion, glucose, fructose, and sucrose levels significantly decreased at 1 DAG compared to 0 DAG. Although their concentrations increased from 3 to 10 DAG across all treatments, LS/HR and HS/HR slightly declined after 7 DAG (Fig. 4I–K). Starch content in the scion steadily increased in all treatments up to 7 DAG, followed by a slight decline in HS/HR and LS/HR at 10 DAG, whereas it continued to rise in HS/LR and LS/LR (Fig. 4L).

In the rootstock, glucose, fructose, and sucrose contents were initially higher at 0 DAG in HS/HR and LS/HR. Glucose and fructose levels gradually declined over time (Fig. 4M and N), whereas sucrose content slightly increased before gradually decreasing and stabilizing at 7 DAG (Fig. 4O). By 10 DAG, all three sugars had declined and stabilized in HS/HR and LS/HR. In contrast, HS/LR and LS/LR consistently exhibited lower sugar levels throughout the healing period, suggesting limited carbohydrate accumulation or mobilization under low-light conditions (Fig. 4).

For rootstock starch, HS/HR and LS/HR had higher initial starch content at 0 DAG but exhibited a marked decline at 1 DAG, while HS/LR and LS/LR remained relatively stable. Starch levels slightly decreased across all treatments and stabilized at 10 DAG. However, HS/HR and LS/HR consistently maintained higher starch contents than HS/LR and LS/LR, highlighting the positive effect of high light intensity on starch accumulation during graft healing (Fig. 4P).

### WGCNA identifies co-expression modules correlated with light-regulated graft healing

**Figure 5:**
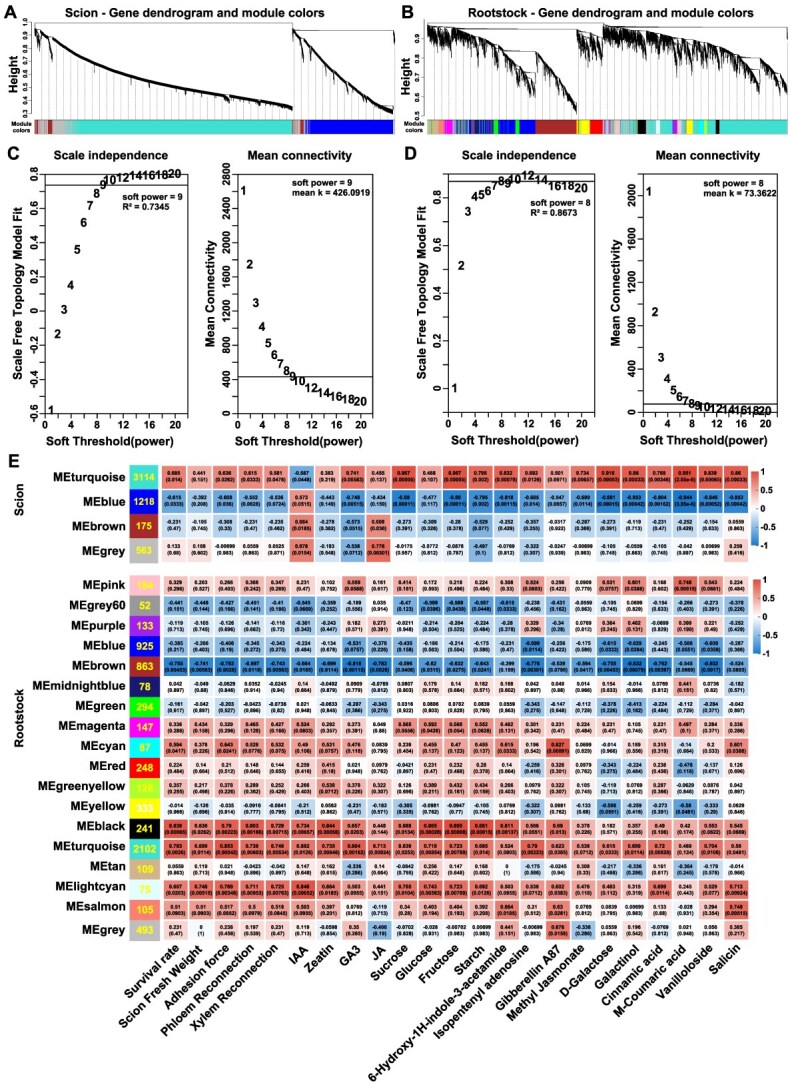
WGCNA of scion and rootstock transcriptomes identifies co-expression modules associated with graft healing under pre-grafting light intensity. A-B, Gene dendrograms of the scion (A) and rootstock (B) generated by WGCNA, showing distinct co-expression modules, each representing a cluster of genes with similar expression patterns. C-D, Scale-free topology and mean connectivity analysis for selecting the soft-thresholding power (β) of 9 for scion (C) and (β) of 8 for rootstock. (D), ensuring the identification of robust co-expression modules. E, Module–trait relationships showing Pearson correlation coefficients between gene co-expression modules and key graft healing traits, including survival rate, adhesion force, phloem and xylem reconnection, as well as hormones, carbohydrates, and phenylpropanoid-related metabolites. Each cell displays the correlation value and its *P*-value, with the strength and direction of correlation indicated by the numerical values and color scale provided in the figure.

To identify gene expression patterns related to graft healing under different light intensities, weighted gene co-expression network analysis (WGCNA) was conducted separately for DEGs in watermelon scion and pumpkin rootstock. Gene clustering identified distinct co-expression modules, visualized as colored branches in the dendrograms (Fig. 5A and B). The appropriate soft-thresholding power (β) was determined based on scale-free topology criteria, with β = 9 selected for the scion network (*R*^2^ = 0.7345; mean connectivity = 426.1) and β = 8 for the rootstock network (*R*^2^ = 0.8673; mean connectivity = 733.4) (Fig. 5C and D).

In the scion, the MEturquoise module (3114 genes) showed strong positive correlations with graft healing traits (Fig. 5E, Table S8), including survival rate (*r* = 0.68), adhesion force (*r* = 0.63), and phloem reconnection (*r* = 0.61), as well as key metabolites such as hormones derivatives (IAA, GA3, JA, and methyl jasmonate), sugar metabolites (sucrose, fructose, starch, D-galactose, and galactinol), and phenylpropanoid-related metabolites involved in lignin biosynthesis (cinnamic acid, m-coummaric acid, and vanilloloside) and stress-related metabolites (salicin). In contrast, the MEblue module (1218 genes) showed negative correlations with these traits and metabolites.

In the rootstock, the MEturquoise module (2102 genes) showed strong positive correlations with graft healing traits (Fig. 5E; Table S8), including survival rate (*r* = 0.78), adhesion force (*r* = 0.85), and phloem and xylem reconnection (*r* = 0.73 and *r* = 0.74, respectively). This module was also positively correlated with several hormone metabolites (IAA, zeatin, and JA), sugar metabolites (sucrose, starch, and galactinol), and lignin-related metabolites (cinnamic acid). Similarly, the MEblack (241 genes) and MElightcyan (75 genes) modules exhibited positive correlations with graft healing traits and associated metabolites. In contrast, the MEbrown module (863 genes) showed negative correlations with most graft healing traits and several key metabolites.

**Figure 6:**
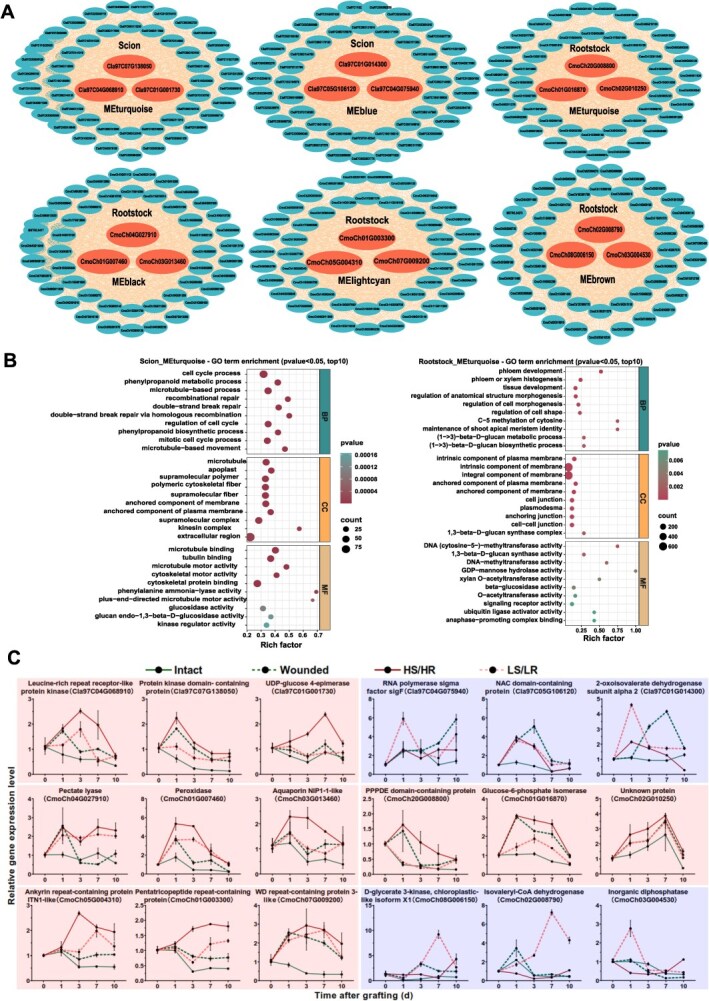
Identification of hub genes in scion and rootstock modules associated with graft healing. A, Hub-gene networks for six key modules identified by WGCNA; turquoise and blue in the scion, turquoise, black, light cyan, and brown in the rootstock. Each network displays the top three hub genes (red) and their most highly connected neighbors (blue). B, Gene Ontology (GO) enrichment analysis for the MEturquoise modules in both scion and rootstock showing the top 10 enriched terms (*P* < 0.05). C, Relative expression of the top three hub genes in each module under four treatment conditions (HS/HR, LS/LR, Wounded, and Intact). Genes showing positive or negative correlations with graft healing traits are indicated in the figure. Data represent mean ± SE (*n* = 3).

To identify key regulatory genes involved in graft healing, hub genes were extracted from WGCNA modules significantly correlated with graft success traits. Network visualization of the top hub genes from modules in both watermelon scion and pumpkin rootstock revealed tightly co-expressed gene clusters (Fig. 6A, Table S9).

GO enrichment analysis of the scion MEturquoise module revealed strong enrichment in cell cycle process, microtubule organization, and phenylpropanoid metabolism, indicating active cell division, cell wall remodeling, and lignin biosynthesis during graft healing (Fig. 6B). GO enrichment of the rootstock MEturquoise module further supported its functional importance, showing significant enrichment in phloem development, xylem differentiation, anatomical structure morphogenesis, plasma membrane components, cell junctions and glucan synthase activity, all critical for vascular reconnection (Fig. 6B). Collectively, these findings indicate that MEturquoise modules in both scion and rootstock are key regulators of gene expression, signal transduction, and structural regeneration associated with callus formation and vascular reconnection during graft healing. Notably, hub genes within these modules included known regulators of cell division (e.g. *CyclinB 1;2*, *CDKB2:1*, and *NACK1*), vascular development (e.g. *PXY*, *WOX4*, and *NAC086*), cell wall remodeling (e.g. *CALS7* and *GH9B3*), hormone signaling (e.g. *IAA5*, *AUX1*, *ARF6*, and *CRE1*) sugar response (e.g. *APL3* and *GDH1*), and phenylpropanoid biosynthesis (e.g. *PAL* and *COMT*), suggesting that successful graft union formation depends on coordinated activation of these pathways under optimal light conditions (Fig. S10).

Genes in the same co-expression module often share function. Thus, if a module is enriched for graft-related processes (auxin signaling, cell wall biogenesis, vascular reconnection, etc.), uncharacterized genes in that module may represent promising novel candidates. Within those six modules, we picked the top three highest connectivity hub genes in each module as novel candidates for grafting healing. Then assessed their expression patterns via RT-qPCR using samples from four experimental conditions, HS/HR, LS/LR, wounded, and intact plant during graft healing (Fig. 6C). We identified 11 novel hub genes (*Cla97C04G068910, Cla97C07G138050*, *Cla97C01G001730*, *CmoCh20G008800*, *CmoCh01G016870*, *CmoCh02G010250, CmoCh04G027910*, *CmoCh01G007460, CmoCh03G013460, CmoCh05G004310*, and *CmoCh01G003300*), all previously unreported in watermelon and pumpkin grafting or generally.

### Integration analysis of transcriptome and metabolome profiles reveals the importance of hormone signaling, carbohydrate metabolism, and lignin biosynthesis during graft union formation

**Figure 7:**
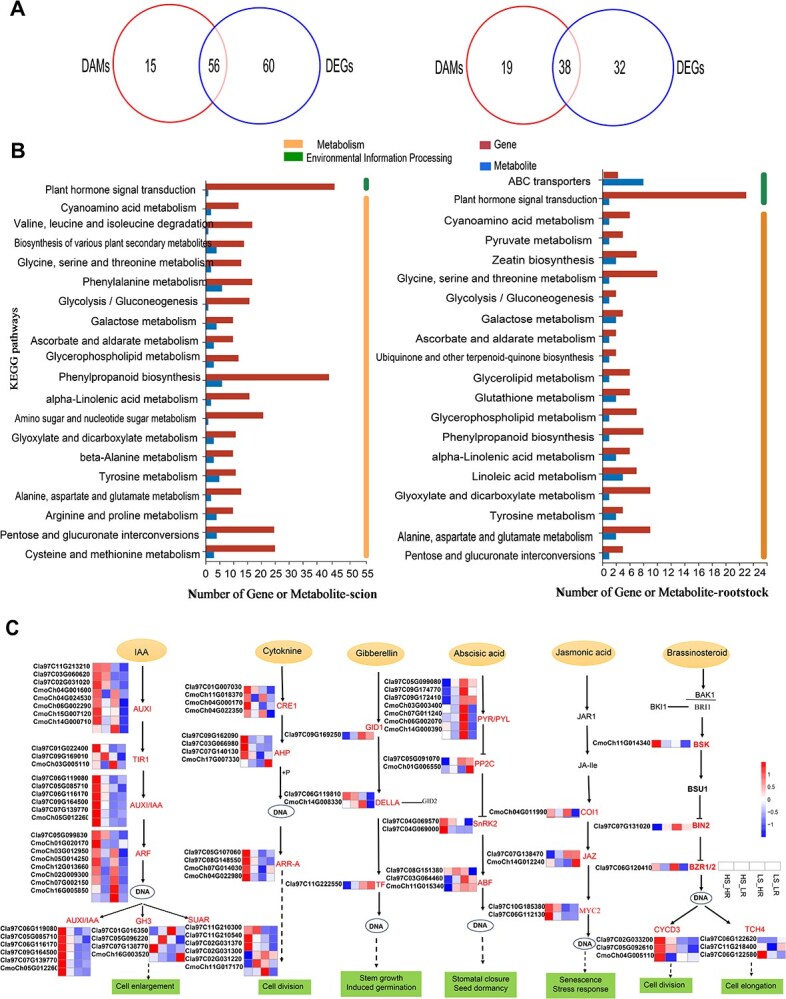
Integration analysis of DEGs and DAMs profiles reveals the importance of hormone signaling during graft union formation. A, Venn diagram shows the overlapping between DEGs and specific DAMs for scion and rootstock (HS/HR vs. LS/LR comparison). B, KEGG enriched of overlapping DEGs and DAMs in scion and rootstock. C, Analysis of DEGs and DAMs associated with hormonal signal transduction pathway for scion and rootstock including auxin (IAA), cytokinin (CKs), gibberellin (GA), abscisic acid (ABA), jasmonic acid (JA), and brassinosteroid (BR). The gene modules involved are indicated by highlights. The rectangular box represents differential gene expression levels of the HS/HR, HS/LR, LS/HR, and LS/LR treatments from left to right by squares sequentially. The heatmap indicates relative expression intensity, with higher and lower expression levels represented according to the scale provided in the figure.

Integrative analysis of DEGs and DAMs revealed convergence at the pathway level, with consistent enrichment in hormone signal transduction, carbohydrate metabolism, and phenylpropanoid biosynthesis (Fig. 7). Their coordinated regulation of genes and metabolites within the same pathways highlights a unified molecular framework underlying graft healing under high light in watermelon seedlings. As shown in Fig. 7A, the Venn diagram for the HS/HR vs. LS/LR comparison indicated 56 and 38 overlapping DEGs and DAMs in the scion and rootstock, respectively. KEGG pathway analysis identified enrichment in plant hormone signal transduction, phenylpropanoid biosynthesis, and carbohydrates metabolism (Fig. 7B), highlighting their coordinated roles in graft union formation.

In these pathways, gene and metabolite profiles analysis indicated significant variations in key genes across different light-intensity treatments (HS/HR, HS/LR, LS/HR, and LS/LR) in both the scion and rootstock. A total of 96 DEGs across 31 gene groups involved in six hormone signaling pathways were identified postgrafting (Fig. 7C; gene annotations in Table S10). Auxin- and cytokinin-related genes were upregulated in watermelon scion and pumpkin rootstock of HS-HR treatment. In the auxin pathway, *AUX/IAA*, *AUX1*, *TIR1*, *ARF*, *SAUR*, and *GH3* genes were consistently upregulated, while cytokinin-related DEGs (*CRE*, *AHP*, and *A-ARR*) showed predominant upregulation. *GID1* and *TF* were upregulated in scion in gibberellin signaling, whereas *DELLA* was upregulated in both species. Abscisic acid-related *PYL* and *ABF* were upregulated in both species, while *SnRK2* was upregulated only in watermelon. In jasmonic acid signaling, *MYC2* was upregulated in watermelon, *COI1* and *JAZ* were upregulated in pumpkin. In the brassinosteroid pathway, *BSK* and *BZR1/2* were upregulated in pumpkin, *BIN2* and *TCH4* were upregulated in watermelon (Fig. 7C).

Phenylpropanoid biosynthesis, crucial for lignin formation, played a key role in graft healing. Lignin biosynthesis-related genes were upregulated early after grafting, indicating their role in vascular reconnection.

The DEGs enriched in *PAL*, *CYP73A*, *4CL*, and *CCR* were observed during graft healing in both watermelon and pumpkin, whereas *COMT*, *CSE*, and *CAD* were upregulated in watermelon, and *FSH*, *TOGT1*, and peroxidase (EC 1.11.1.7) were upregulated in pumpkin (Fig. S11, Table S10).

After grafting, the integrated analysis of DEGs and DAMs revealed significant enrichment of the starch and sucrose metabolism pathway. Fifty-four DEGs involved in soluble sugar metabolism pathways were identified in both watermelon and pumpkin, with most being upregulated in the HS-HR treatment (Fig. S12, Table S10). The annotation and FPKM values of these DEGs are summarized in Table S8. The expression of genes encoding invertase, trehalose-6-phosphate phosphatase, sucrose synthase, and hexokinase (*INV*, *TPS*, *SUS*, and *HK*) was upregulated in both watermelon and pumpkin. These enzymes play a crucial role in carbohydrate metabolism and energy regulation. Conversely, *GN1*, *GN4*, and *bglB* were upregulated in watermelon, while *UGP2* and *SPP* were upregulated in pumpkin (Fig. S12).

## Discussion

Splice grafting without rootstock cotyledons presents a promising alternative by eliminating meristematic tissues responsible for regrowth, potentially reducing grafting costs [7]. However, the absence of rootstock cotyledons has been associated with grafting failure [7], necessitating further research to enhance survival and seedling quality.

Artificial light intensity is crucial in producing high-quality vegetable seedlings, with cucumber and tomato seedlings benefiting from moderate to high light intensities, a light intensity of 240–300 μmol·m^−2^·s^−1^ optimizes seedling growth, enhancing root and shoot dry weight, stem diameter, and photosynthesis rate, yielding results comparable to greenhouse conditions [36]. Customizing light conditions to meet the specific requirements of each crop can improve growth, quality, and energy efficiency in plant factories. PPFD of 260 μmol·m^−2^·s^−1^ has been found effective for cucumber scion and rootstock production in plant factories with artificial light, improving plant biomass, seedling health index, compactness, and quality indices [37]. Similarly, Hwang *et al.* [27] further demonstrated that increasing light intensity from 100 to 300 μmol·m^−2^·s^−1^ resulted in the broadest leaf area in tomatoes and red peppers, emphasizing the critical role of light in promoting seedling vigor. Our findings revealed that a high light intensity of 300 μmol·m^−2^·s^−1^ for watermelon scion and pumpkin rootstock prior to grafting significantly enhanced the survival rate and seedling quality of cotyledon-less splice-grafted watermelon seedlings (Fig. 1). This improvement can be attributed to the positive effects of high light intensity on seedling physiology, which promotes photosynthetic activity and carbon assimilation, enhances metabolic activation, carbohydrate metabolism, and hormonal regulation, leading to development of robust and vigorous tissues. These stronger tissues are more resilient and better equipped to establish a successful graft union.

Graft healing is a critical yet complex developmental process in plant grafting. Recent studies have increasingly highlighted specific metabolites as early indicators of successful graft union formation. Branched-chain amino acids, basic amino acids such as asparagine, and stilbene compounds, particularly trans-ε-viniferin, accumulated at the graft union, supporting callus formation and graft development [38, 39]. Key metabolites involved in graft union formation include secondary metabolites, mainly phenolic compounds such as polyphenols and flavonoids, which contribute to graft healing by enhancing defense responses, promoting cell division, and facilitating tissue differentiation, ultimately supporting the establishment of a stable graft union [40]. Our metabolomics analysis revealed a higher abundance of metabolites related to hormones, carbohydrate derivatives and phenylpropanoid biosynthesis such as 6-hydroxy-1H-indole-3-acetamide, trans-zeatin, D-galactose, galactinol, and m-coumaric acid, as well as cinnamic acid, under high light intensity during graft healing (Fig. 2).

RNA sequencing (RNA-seq) is a powerful tool for investigating transcriptional dynamics and regulatory mechanisms during grafting [41, 42]. Our RNA-seq analysis revealed that plant hormone signal transduction, phenylpropanoid biosynthesis, phenylalanine metabolism, and starch and sucrose metabolism were overlapping pathways identified across different grafting combinations in response to varying light intensities (Fig. 3). Previous studies have demonstrated the essential roles of plant hormone signal transduction, phenylpropanoid biosynthesis, and phenylalanine metabolism in graft healing [43]. Functional categorization of DEGs indicated that graft healing activated a large number of transcripts involved in plant hormone signaling, phenylpropanoid biosynthesis, carbon metabolism, and wound responses [44]. Additionally, we identified numerous genes associated with graft healing, including those involved in cell division, cell wall modification, cambium development, and phloem and xylem formation, which were significantly upregulated under high light intensity for scion and rootstock compared to low light (Fig.S7B). These findings are consistent with studies on heterografting, where genes related to auxin signaling (*IAA1*), wound repair (*ANAC071*), and cambial and vascular development (*GH9B3*, *EXPB3*, and *OPS*) were upregulated in response to interfamily heterografting in *Nicotiana benthamiana* and *Arabidopsis thaliana* [45]. Grapevine, Arabidopsis, and tomato studies have identified conserved regulatory pathways related to cell wall modification and vascular development essential for graft union formation [46, 47]. Key processes include the upregulation of genes involved in cambium development, cell division, phloem and xylem formation, and secondary metabolism [48]. Previous studies have identified genes associated with cell division and vascular reconnection, including *Histon H4*, *Cyclin B1;2*, *WOX4*, *TMO6*, *PXY*, and *VND7*, which were upregulated in graft junctions compared to intact plants [41]. Thousands of DEGs involved in cambium development, cell division, and phloem and xylem differentiation (*WOX4*, *PXY*, *ATHB8*, *SMXL4*, and *SMXL5*) were identified in graft junctions of Norway spruce and Arabidopsis during grafting [16]. These findings provide significant insights into the molecular mechanisms underlying successful graft healing and vascular reconnection in response to high light intensity.

Our WGCNA identified distinct gene modules in both scion and rootstock that were strongly associated with graft healing traits and light-responsive metabolic changes. The MEturquoise modules in both scion and rootstock exhibit strong positive correlations with graft healing traits such as survival rate, adhesion strength, and vascular reconnection, as well as metabolite accumulation including, hormones, and lignin precursors (Fig. 5). This aligns with previous findings that auxin signaling, sugar, and lignin biosynthesis genes are coordinately activated at the graft union [48–50]. Additionally, hub gene network analysis revealed several novel candidate regulators (e.g. *Cla97C04G068910, Cla97C07G138050*, *Cla97C01G001730*, *CmoCh20G008800*, *CmoCh01G016870*, *CmoCh02G010250, CmoCh04G027910*, *CmoCh01G007460, CmoCh03G013460, CmoCh05G004310*, *CmoCh01G003300*; Fig. 6). These genes related to carbohydrate metabolism, cell wall remodeling, tissue adhesion, and signal transduction, previously unreported in grafting contexts, emphasizing the predictive power of WGCNA in discovering functionally relevant genes.

Plant hormones play a crucial role in vascular bundle reconnection during grafting. Our results identified multiple genes and metabolites involved in hormone signal transduction pathways, with most auxin-responsive genes exhibiting significant transcriptional changes during graft healing under varying light-intensity treatments (Fig. 7). This finding aligns with previous studies demonstrating the importance of auxin-response genes in grafting [41]. Auxin facilitates graft union formation by upregulating genes involved in tissue attachment, cell proliferation, and vascular reconnection in both rootstock and scion tissues [51]. Additionally, differential accumulation of auxin and cytokinin above and below the graft junction promotes xylem and phloem patterning during tomato graft formation [22]. The roles of various plant hormones, including auxin, gibberellin, abscisic acid, jasmonic acid, and cytokinin, differ in scion and rootstock tissues during graft healing [52].

As graft wound healing progresses, the activities some antioxidant enzymes decline while those involved in lignin biosynthesis increase [53]. Our analysis revealed significant upregulation of genes and metabolites associated with phenylpropanoid biosynthesis, particularly lignin biosynthesis genes such as *PAL*, *CYP73A*, *4CL*, and *COMT* (Fig. S11). This aligns with a recent study demonstrating the crucial role of the lignin biosynthesis pathway in vascular bundle formation during cucumber grafting, with increased light intensity leading to the upregulation of *COMT* and *CAD* genes over time [50].

High light intensity for both scion and rootstock significantly influence DEGs and DAMs involved in carbohydrate metabolism pathways in watermelon grafted seedlings without rootstock cotyledons (Fig. S12). Increased light intensity enhances photoassimilate production, facilitating the translocation of photosynthates from source organs (cotyledons) to sink organs (hypocotyl), thereby promoting biomass accumulation, and rootstock vitality. Sugars play a crucial role in graft union development by promoting vascular reconnection and growth, with appropriate sugar content being essential for successful grafting, as demonstrated in cucumber/pumpkin heterografts [23]. Studies have shown that increased concentrations of soluble sugars and starch in rootstock seedlings improve grafting survival rates and regulate graft healing [8, 10]. Additionally, heterografting affects sugar distribution between the scion and rootstock after graft establishment, with rootstock cotyledons serving as a buffer in this process [25].

Our study demonstrated that modulating pre-grafting light intensity enhances healing efficiency during the critical early stage of graft union formation, significantly improving the survival rate of cotyledon-less grafted watermelon transplants. This provides a practical strategy to overcome the limitations associated with the absence of rootstock cotyledons during grafting. This early healing period is pivotal for ensuring successful graft healing and vigorous grafted seedling establishment, which are known essential for improved yield and fruit quality later [54, 55]. Graft survival positively correlated with later yield, as only plants that successfully survive during the healing process can contribute to the final crop yield. High survival rates increase the number of productive plants, thereby directly enhancing overall fruit yield and quality [11]. Future research will build on these findings to evaluate how enhanced early healing and survival impact long-term field performance, including yield and fruit traits.

**Figure 8:**
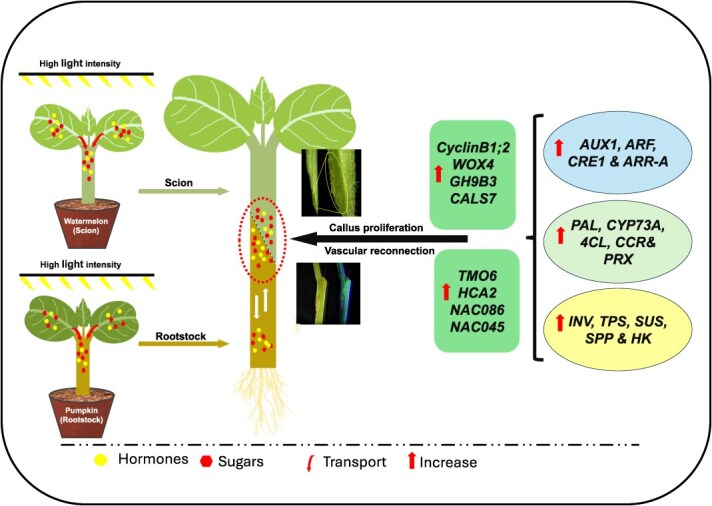
A proposed model illustrating the effect of high light intensity on the graft union formation and vascular reconnection of cotyledon-less grafted watermelon transplants. High light intensity promotes translocation and storage of hormones and sugars in hypocotyls of scion and rootstock inducing hormone signaling (*AUX1*, *ARF*, *CRE1*, and *ARR-A*), phenylpropanoid biosynthesis enzymes (*PAL*, *CYP73A*, *4CL*, *CCR*, and *PRX*), and carbohydrate metabolism related genes (*INV*, *TPS*, *SPP*, *SUS*, and *HK*). These activate cell division, cell wall modification, and phloem and xylem related gene (e.g., *Cyclin B1;2*, *WOX4*, *GH9B3*, *TMO6*, and *HCA2*), driving callus proliferation and vascular reconnection to improve grafting success.

In conclusion, our findings provided a novel regulatory mechanism of high light intensity in graft union formation and root regeneration of cotyledon-less grafted watermelon seedlings (Fig. 8). High light intensity pre grafting enhances the translocation and storage of hormones and sugars in the hypocotyl of scion and rootstock. High-light conditions induce transcriptional and metabolic alterations related to hormone signaling, phenylpropanoid biosynthesis enzymes, and carbohydrate metabolism. Collectively, these changes induce cell division related genes, leading to rapid callus proliferation, modulate cell wall modification genes to improve tissue adhesion, and strengthen vascular reconnection by early activation phloem and xylem development related genes leading to a more robust graft union and improved seedling survival. Future research should investigate optimal light spectra and intensity thresholds to further refine grafting technologies for large-scale applications.

## Materials and methods

### Plant growth conditions and light treatments

The experiment was carried out at the National Center of Vegetable Improvement, Huazhong Agricultural University, Wuhan, Hubei, China (latitude 30°27’ N, longitude 114°20′ E). Watermelon cultivar ‘Zaojia 8424’ (*C. lanatus*) was used as the scion, while the pumpkin hybrid ‘Qingyu No. 1’ (*C. maxima × C. moschata*) was the rootstock. Scion and rootstock seeds were sown in 128-cell and 50-cell trays filled with seedling substrate, respectively. Seeds were germinated in a controlled environment under darkness for 48 h at 25–28°C with 90% relative humidity. Scion seeds were sown two days after the rootstock seeds to synchronize their growth for grafting, following standard commercial nursery practices. After germination, watermelon scion and pumpkin rootstock seedlings were grown in a PFAL for 8 and 10 days, respectively, under a 14/10-h light/dark photoperiod using white LED illumination, with relative humidity maintained at 60%–70%. Throughout the growing period, plants were watered and fertilized with a 1:1000 diluted water-soluble fertilizer (20-10-20 + TE, Hubei Greencare Agriculture Co., Ltd., Wuhan, China).

The PFAL exterior was constructed with a 70-mm urethane foam insulation to protect against environmental fluctuations. The interior featured an air conditioning unit to regulate temperature and ensure air circulation, along with a seedling module and an environmental control system. Each seedling module contained six 35 W white LEDs (YN-MV35WD, Shenzhen NN lighting Co., Ltd., China) positioned above the seedbed, with light intensity, photoperiod, and other parameters controlled via software. PPFD intensities of 100 and 300 μmol·m^−2^·s^−1^, with a 14/10-h light/dark photoperiod, were applied to evaluate the growth response of watermelon scions and pumpkin rootstocks under different light conditions.

All light-intensity treatments were defined by both spectral composition and PPFD using a spectrometer (OHSP-350S, HopooColor Technology Co., Ltd., Zhejiang, China). The broad-spectrum white LEDs exhibited dominant emissions in both blue and red regions, red:blue ratio (3:2) (Fig. S13). PPFD measurements across the seedling growth module demonstrated high uniformity (>95%), as detailed in Supplementary Fig. S14.

### Methods of grafting and healing

Grafting was performed using the splice method without rootstock cotyledons, as described by Devi *et al.* [7], coinciding with the emergence of the scion’s first true leaf and the unfolding of the rootstock’s first true leaf. The rootstock and scion were cut at a 60° angle, 0.5 cm and 2 cm below the cotyledons, respectively. To ensure a stable graft union, the cut surfaces were aligned and secured with a watermelon grafting clip (2.5–3 mm). The root-excised rootstock was severed at the base of the hypocotyl, just above the soil line, grafted, and then placed approximately 2–3 cm deep into seedling trays containing potting mix [51]. Each treatment included three replicates, with 50 plants per replicate, totaling 150 grafted plants per treatment. Immediately after grafting, all seedlings were enclosed with a transparent plastic cover, placed in a controlled healing chamber, and kept in complete darkness for 1 day. From 2–3 DAG, the seedlings subjected to low light intensity (40 μmol·m^−2^·s^−1^). The light intensity was then increased to 80 μmol·m^−2^·s^−1^ from 4–6 DAG, and further to 150 μmol·m^−2^·s^−1^ from 7 DAG onward. The photoperiod was maintained at 14/10 h (light/dark) per day. Daytime and nighttime temperatures were set at 28 and 18°C, respectively. Humidity was maintained above 95% for the first five days and gradually reduced to 85% from days 6 to 10. Following standard practice, the plants were removed from the healing chamber on day 11 and transferred to the growth room.

### Determination of survival rate and growth index of grafted plants

The survival rate was assessed 14 DAG using the formula:


\begin{align*} \mbox{Survival rate (\%)}= \mbox{(Number of surviving grafted seedlings/}\\ \mbox{Total number of grafted 720 seedlings)} \times 100 \end{align*}

Surviving grafted plants exhibited healthy scion leaves and rootstock stems, while wilting indicated graft failure. Five grafted plants per replicate, totaling fifteen plants per treatment, were analyzed for growth indices, including fresh weight, length, stem diameter, and leaf area of the watermelon scion. Leaf area was measured by photographing the leaves of each plant and calculating the area using ImageJ software (available at https://imagej.nih.gov/ij/).

The root systems of grafted seedlings were washed with distilled water, and their morphology was imaged using a root scanning system (Epson, UE-88). Root growth parameters, including total root length, diameter, volume, and surface area, were analyzed using WinRHIZO software (Quebec, QC, Canada).

### Assessment of adhesion force and vascular connectivity

Samples were collected 1, 3, 5, and 7 DAG. Twenty grafted plants were analyzed per replicate, with three replicates each time. The adhesive force between the scion and rootstock was quantified using a tensiometer (ZTS-5 N). After removing the grafting clip, the rootstock was attached to the tensiometer’s hook using a suspended clip, securing the lower end of the rootstock stem. The scion was then pulled horizontally until detachment, and the force was recorded.

Phloem and xylem connections were assessed using carboxyfluorescein diacetate (CFDA) and acid fuchsin transport across the graft union, respectively. For phloem reconnection, CFDA (1 mM) was applied to wounded scion cotyledons after incision with sandpaper, and fluorescence in the rootstock hypocotyl was assessed after 40 min at 26°C under dark conditions [51]. For xylem connectivity, seedling roots were washed and immersed in 5 ml of 0.1% acid fuchsin, and fluorescence in the scion hypocotyl was analyzed after 40 min under dark conditions [56]. Fluorescence signals were detected using a stereoscopic fluorescence microscope (Leica M205FA, Leica, Germany), and reconnection rates were calculated using the formula:


\begin{equation*} \mbox{Reconnection rates = fluorescence signal plants/total plants} \times 100 \end{equation*}

Fluorescence signals in the scion were detected under GFP using a stereoscopic fluorescence microscope (Leica M205FA).

### Metabolic profiling

The plant samples were collected from graft union tissue of the scion and rootstock. A 0.5-cm segment was collected 24 h after grafting to evaluate the early response of pre-grafting light intensity on grafting success. Forty plants were randomly selected per treatment, with every ten combined to form one replicate. The collected samples were used for nontargeted metabolomics analysis. Briefly, 100 mg of tissue was dissolved in 400 μl of extraction solution (methanol: water, 4:1, v/v) with 0.3 mg/ml L-2-chlorophenylalanine as an internal standard. The samples were maintained at −10°C, homogenized (50 Hz, 6 min), stored at −20°C for 30 min, centrifuged (13 000 rpm, 4°C, 15 min), and the supernatants were transferred to vials for detection. Equal amounts of each sample were pooled to create QC samples, which were injected regularly to ensure data consistency.

Metabolite separation was conducted using a Thermo UHPLC-Q Exactive system with solvent A (0.1% formic acid in acetonitrile: water, 95:5, v/v) and solvent B (0.1% formic acid in acetonitrile: isopropanol: water, 47.5:47.5:5, v/v). Raw data were analyzed using Progenesis QI (Waters Corporation, USA), generating a three-dimensional data matrix (CSV) containing sample details, metabolite IDs, and spectral intensities.

PCA was performed to identify variations in DAMs between the samples. DAMs were selected based on VIP > 1 (OPLS-DA model) and *P* < 0.05 (*t*-test). DAMs were mapped to enrichment pathways using the KEGG database (https://www.genome.jp/kegg/).

### Transcriptomic analysis

The plant tissues were sampled and prepared as described for the metabolomic analysis. Thirty plants were randomly selected per treatment, with every ten combined to form one RNA-seq sample, yielding three biological replicates.

Total RNA was extracted, and its concentration and purity were assessed using a NanoDrop 2000 (Thermo Scientific, USA) and a 2100 Bioanalyzer (Agilent Technologies, USA), respectively. High-quality RNA samples (RQN > 6.5, OD260/280 = 1.8–2.2, concentration ≥ 30 ng/μl) were used for sequencing library construction. A total of 24 libraries were generated and sequenced using the Illumina HiSeq X Ten/NovaSeq 6000 system (2 × 150 bp read length) at Shanghai Majorbio Biological Technology Co., Ltd. After removing low-quality reads, approximately 6.25 Gb of clean reads per sample were aligned to the *C. moschata* (Rifu) and *C. lanatus* (97103) v2 genomes using HISAT2. The aligned reads were assembled using StringTie, and expression levels were quantified using fragments per kilobase of exon per million reads (FPKM).

DEG analysis was conducted using the DESeq R package (v1.24.0). Genes with |log2FC| > 1 and q-value <0.05 were considered significantly differentially expressed, including both upregulated and downregulated genes. Gene function annotation and enrichment analyses were performed using the GO and KEGG databases. Data were analyzed via the Majorbio Cloud Platform (www.majorbio.com).

### Determination of phytohormone content

The phytohormone levels of auxins (IAA), cytokinins (CKs), gibberellins (GA), and jasmonic acid (JA) at 0, 1, 3, 7, and 10 DAG were measured according to the method described by Duan *et al.* [57], with slight modifications. Briefly, 0.1 g of finely ground tissue was dissolved in 1 ml of cold extraction buffer (isopropanol:water: hydrochloric acid, 15:4:1, v/v/v) with 10 μl of a mixed internal standard. The mixture was shaken at 4°C for 30 min in darkness, followed by adding 4 ml of dichloromethane and an additional 30 min of shaking. The sample was then centrifuged at 6000 g and 4°C for 15 min, and 4 ml of the lower phase was transferred to a new tube and concentrated using a nitrogen blower without completely drying. The extract was then re-dissolved in 100 μl of 80% methanol (v/v) and centrifuged at 13 000 g and 4°C for 15 min.

The supernatant was analyzed using tandem mass spectrometry (MS/MS, Applied Biosystems 6500 Triple Quadrupole) and ultra-performance liquid chromatography (UPLC, ExionLC™ AD, https://sciex.com.cn/). Data were collected using the Analyst 1.6.3 program (AB Sciex). The hormone content was calculated using the formula: 


\begin{equation*} \mbox{Hormone content } (ng \cdot g^{-1}) = 0.001\ C \times V/m \end{equation*}

where *C* is the concentration from the standard curve (ng·ml^−1^), *V* is the re-dissolving volume (μl), and *m* is the sample weight (g).

### Determination of sugars and starch contents

Sugar levels (glucose, fructose, and sucrose) were analyzed at 0, 1, 3, 7, and 10 DAG using gas chromatography (GC) as described by Kaleem *et al.* [58]. Briefly, 0.1 g of plant tissue was extracted and analyzed with an Agilent 7890B gas chromatograph, fitted with a nonpolar HP-5MS phenylmethylsiloxane column and a flame ionization detector. Starch content was determined using a Starch Content Assay Kit (Solarbio, Cat# BC0700) following the manufacturer’s protocol.

### Identification of hub genes through WGCNA analysis

WGCNA R package (v1.71) used to identify co-expression modules from the watermelon scion and pumpkin rootstock DEGs on the Majorbio Cloud Platform (www.majorbio.com) (Fig. 3). These modules were then correlated with the phenotypic traits (survival rate, scion growth, adhesion force and vascular reconnection) and relative abundances of key DAMs using Pearson correlation coefficients (Fig. 2F). Modules of co expressed genes were identified using dynamic tree cutting with the following parameters; soft-thresholding power (β) of 9, minimum module size of 30, merged module cut height of 0.25, and minimum module membership (KME) to remain in a module of 0.3. A module was considered significant if the absolute correlation *R*^2^ > 0.6 and *P* < 0.05.

In the resulting scale free network, each DEG was represented as a node, and edges were based on Pearson correlation of gene expression profiles between gene pairs. Hub genes within modules were identified by visual network analysis in Cytoscape (v3.3.0) using default thresholds for intra-module connectivity [43] and edge weight (0.02). In the network, larger node sizes indicated genes with more connections and potential regulatory importance. The integration of gene modules with phenotypic traits helped identify key modules and genes associated with graft healing.

### qRT-PCR

To validate RNA-Seq data via qRT-PCR, twelve genes were selected from watermelon and pumpkin. Total RNA was extracted from samples collected as described previously using the TranZol method. cDNA was synthesized using the cDNA Synthesis SuperMix Kit, and qRT-PCR was performed with the SYBR Green qPCR SuperMix Kit (TransGen Biotech Co., Ltd., China) on a QuantStudio 7 Flex Real-Time PCR System (Applied Biosystems, USA). Primers were designed using Primer3Plus (Supplemental Table S2). *β-actin* and *ClADP* were used as reference genes for pumpkin and watermelon, respectively, with relative expression calculated using the 2^-ΔΔCt^ method.

### Statistical analysis

The physiological data were analyzed using Statistix 8.1, while metabolomic and transcriptomic data were processed on the Majorbio I-Sanger Cloud Platform. Figures were generated using GraphPad Prism 8.0 (GraphPad Software Inc., San Diego, CA, USA), and significance was determined by Duncan’s multiple range test (*P* < 0.05).

## Supplementary Material

Web_Material_uhaf293

## Data Availability

The transcriptomics raw data generated in this study have been deposited in the National Center for Biotechnology Information (NCBI) database under accession code PRJNA1155268 for watermelon and PRJNA1155326 for pumpkin.
