# Supplementary material for: Integrated multiomics analysis reveals the molecular mechanism of light intensity-enhanced healing in cotyledon-less splice grafted watermelon
Source: Hortic Res. 2025 Oct 30;13(2):uhaf293. doi: 10.1093/hr/uhaf293 (PMC12923272; doi:10.1093/hr/uhaf293)
Supplement: Web_Material_uhaf293 [file web_material_uhaf293.zip › Supplamentry Figures-R1.docx]

**Integrated multi-omics analysis revealing the molecular mechanism of light intensity-enhanced healing in cotyledon-less splice grafted watermelon**

Yehia Abouseif ^1,2^, Akebaierjiang Kadeer^1^, Cao Haishun^3^, Muhammad Mohsin Kaleem^1^, Michitaka Notaguchi^1,4^, Xie Qifan^5^, Qingjun^5^, Zhilong Bie^1^, Yuan Huang^1*^

^1^National Key Laboratory for Germplasm Innovation and Utilization of Horticultural Crops, College of Horticulture and Forestry Sciences, Huazhong Agricultural University, Wuhan, 430070, Hubei Province, PR China

^2^Horticulture Research Institute, Agricultural Research Center, Giza 12119, Egypt

^3^Institute of Facility Agriculture, Guangdong Academy of Agricultural Sciences, Guangzhou, China

^4^Department of Botany, Graduate School of Science, Kyoto University, Kitashirakawa Oiwake-cho, Sakyo-ku, Kyoto 606-8502, Japan

^5^Guangxi Academy of Sericultural Sciences, Nanning 530007, China

^*^Corresponding authors, E-mail: [huangyuan@mail.hzau.edu.cn](mailto:huangyuan@mail.hzau.edu.cn)


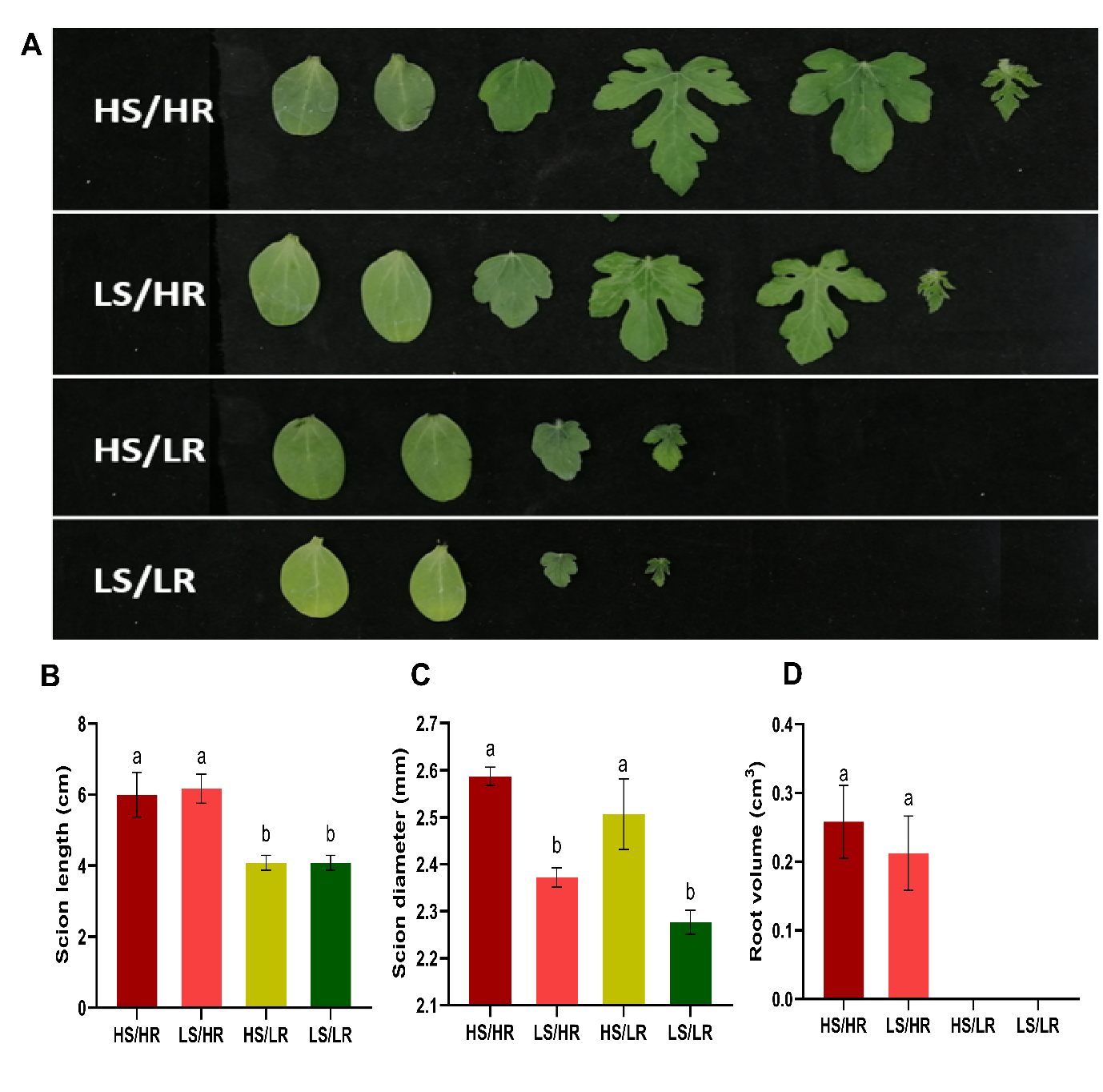


**Fig. S1.** Effect of light intensity before grafting on cotyledons-less grafted watermelon transplants. A, Scion leaf count. B, Scion length (cm). C, Scion diameter (mm). D, Root volume (cm^3^) measured at 14 days after grafting (DAG).





**Fig. S2.** Hierarchical clustering heatmap of differentially accumulated metabolites (DAMs) in scion and rootstock. Colored bars adjacent to the dendrogram indicate major metabolite classes, heatmap highlights distinct accumulation patterns, particularly in hormone, carbohydrate, and phenylpropanoid pathways related metabolites associated with graft healing.





**Fig. S3.** KEGG enrichment analysis of differential accumulated metabolites (DAMs) for scion and rootstock under different light intensities. The color indicates the p-value (from the lowest to the highest), and the bubble size indicates the number of genes.


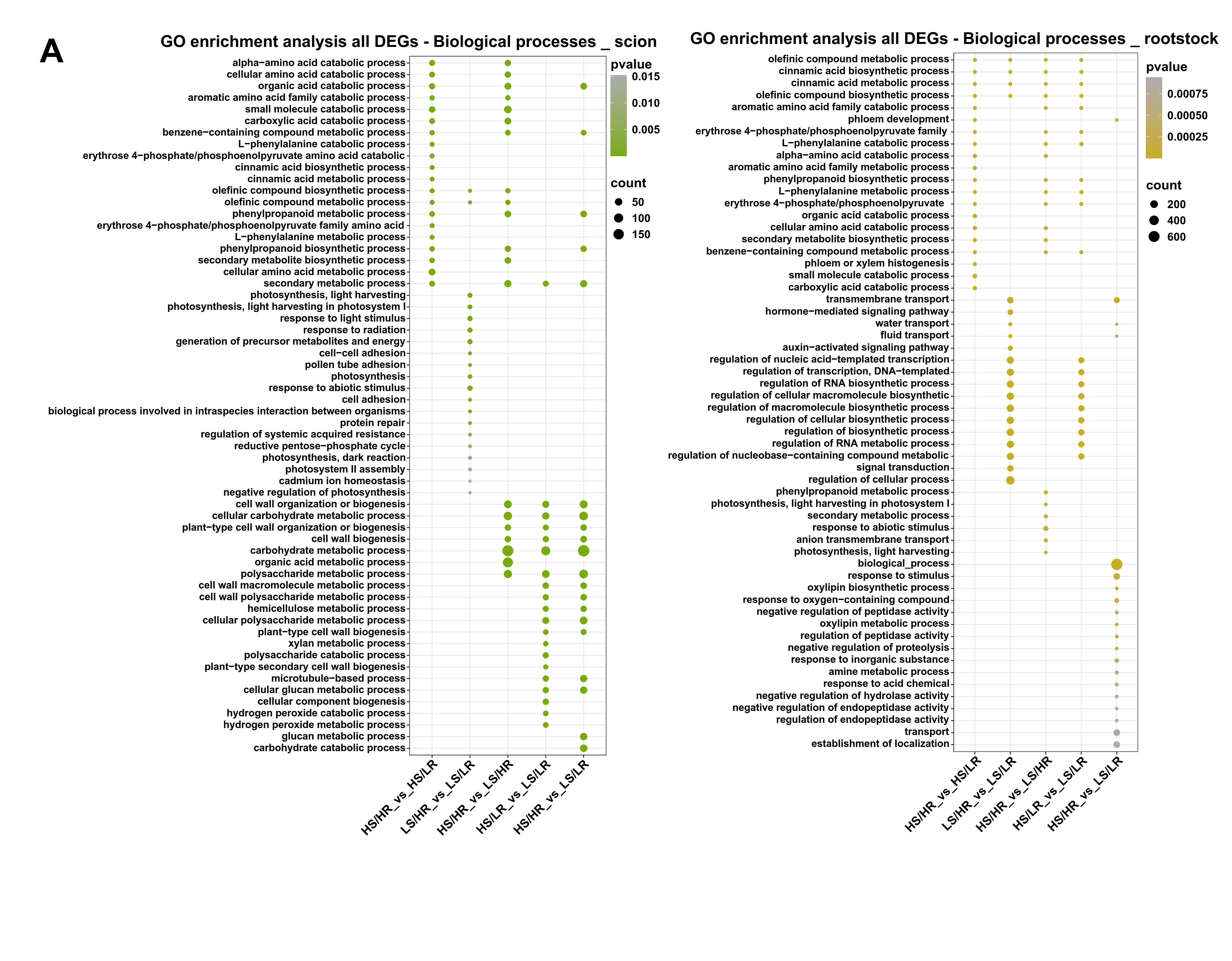


**Fig. S4.** Gene Ontology (GO) enrichment analysis of differentially expressed genes (DEGs) in the scion and rootstock. The top 20 significantly enriched GO biological process (BP) terms are shown. The x-axis represents treatments and the y-axis the significantly enriched GO terms. The color indicates the p-value (from the lowest in red to the highest in blue), and the bubble size indicates the number of genes.


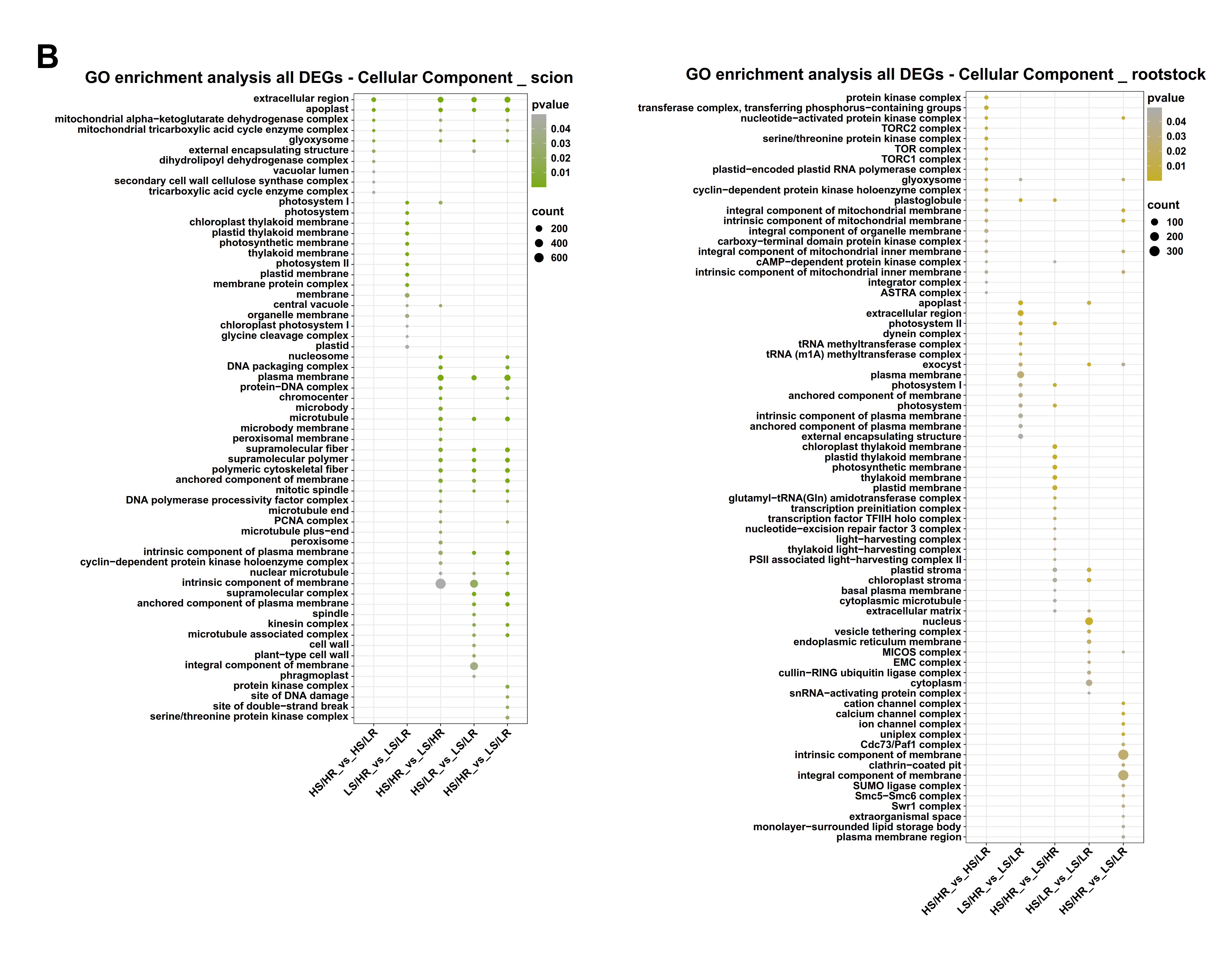


**Fig. S5.** Gene Ontology (GO) enrichment analysis of differentially expressed genes (DEGs) in the scion and rootstock. The top 20 significantly enriched GO cellular component (CC) terms are shown. The x-axis represents treatments and the y-axis the significantly enriched GO terms. The color indicates the p-value (from the lowest in red to the highest in blue), and the bubble size indicates the number of genes.


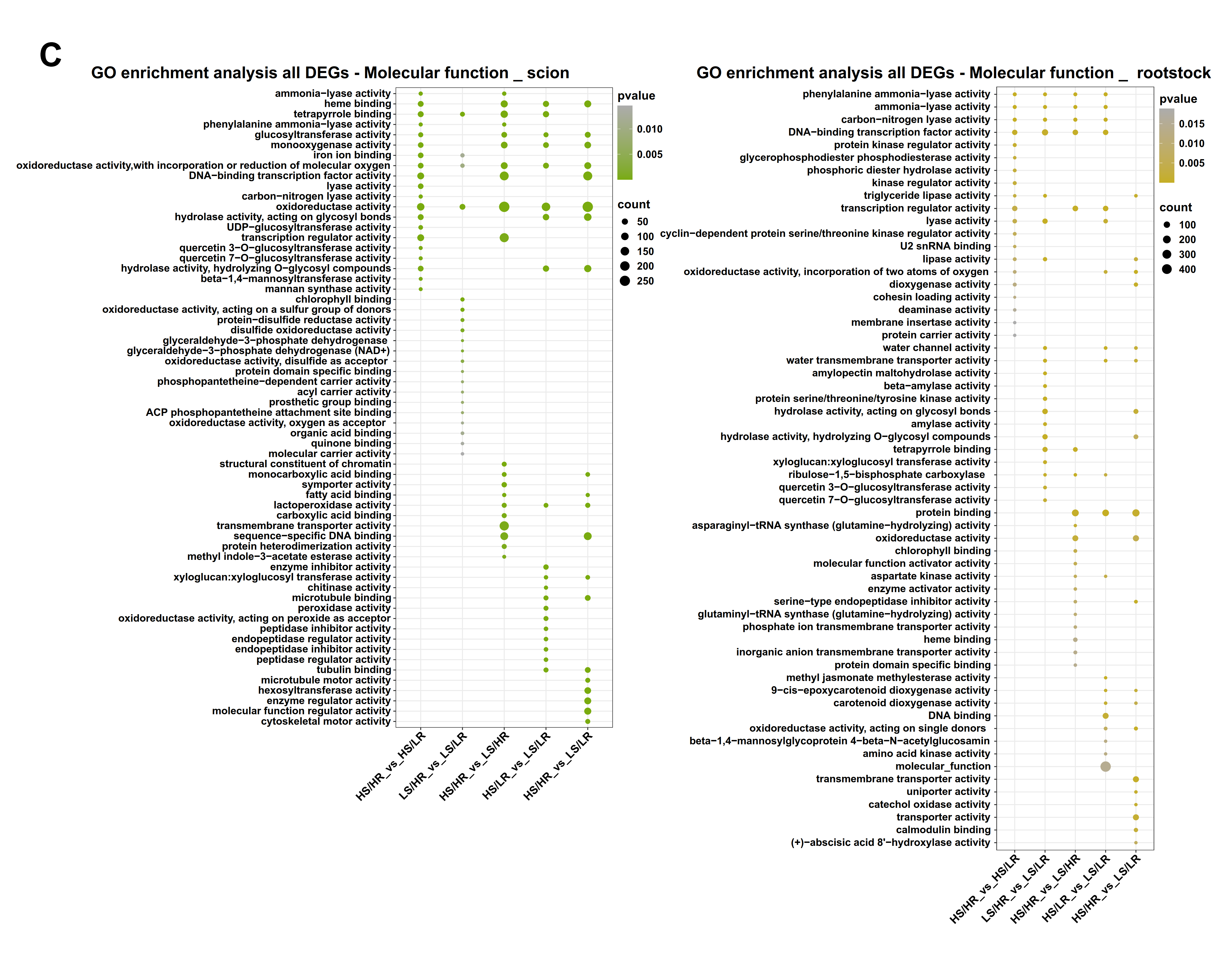


**Fig. S6.** Gene Ontology (GO) enrichment analysis of differentially expressed genes (DEGs) in the scion and rootstock. The top 20 significantly enriched GO molecular function (MF) terms are shown. The x-axis represents treatments and the y-axis the significantly enriched GO terms. The color indicates the p-value (from the lowest in red to the highest in blue), and the bubble size indicates the number of genes.





**Fig. S7.** Light induces tissue-specific transcriptional activation of graft related genes. A, Go enrichment analysis of the top 20 DEG terms in all HS vs LS upregulated genes of the scion and all HR vs LR upregulated genes of the rootstock. B, Heatmap represent expression profiles of graft related DEGs; wound reunion, cell division, cambium, provasculature, xylem, and phloem development in scion and rootstock.


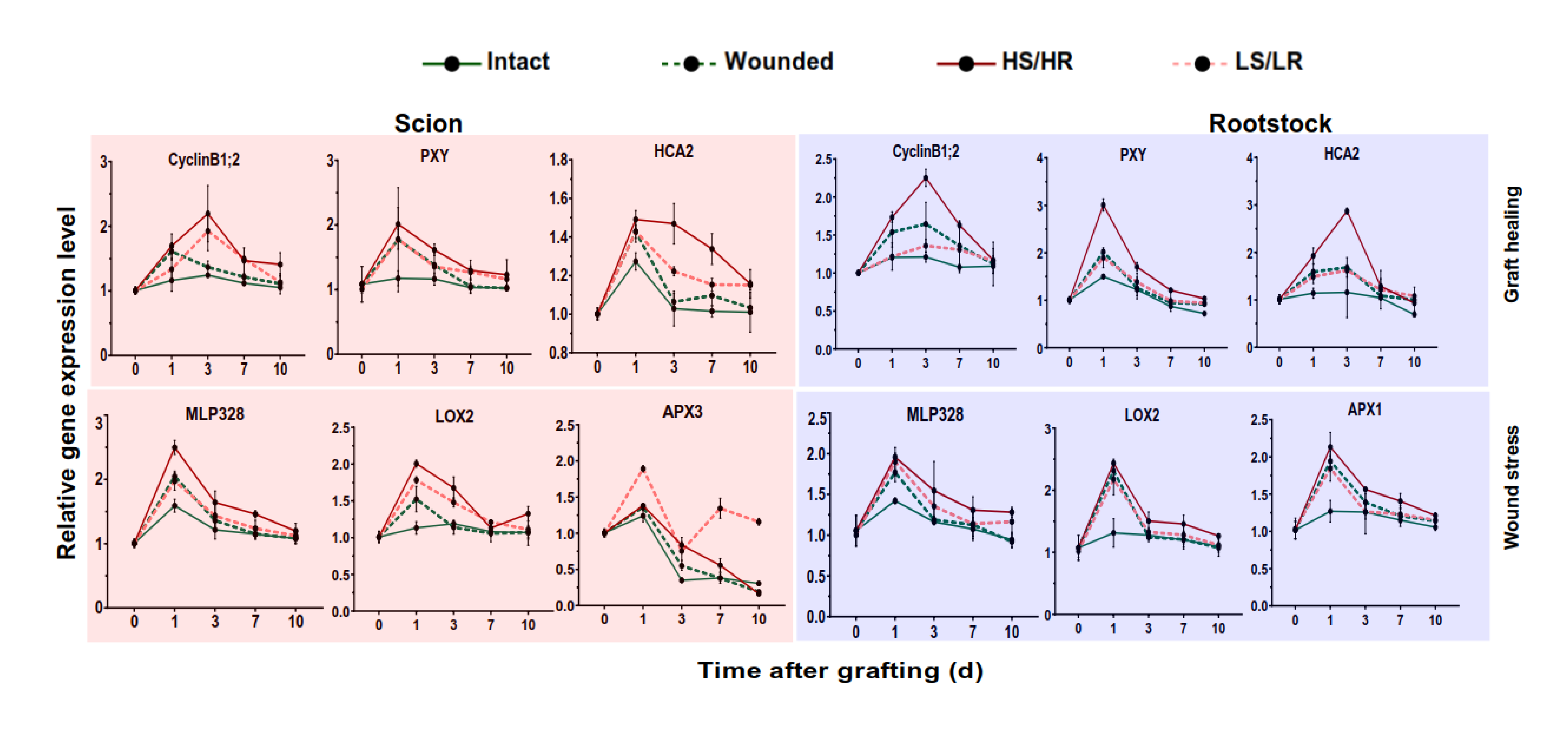


**Fig. S8.** Relative expression profiles of graft healing and wound stress-related genes in scion and rootstock tissues during grafting. Relative gene expression levels of *CyclinB1;2*, *PXY* and *HCA2* (graft healing markers), and *MLP328*, *LOX2* and *APX1/APX3* (wound stress markers) were measured in scion (pink panels) and rootstock (blue panels) tissues during grafting. Four treatment groups are shown; grafting combinations (HS/HR, LS/LR), wounded and intact plants.


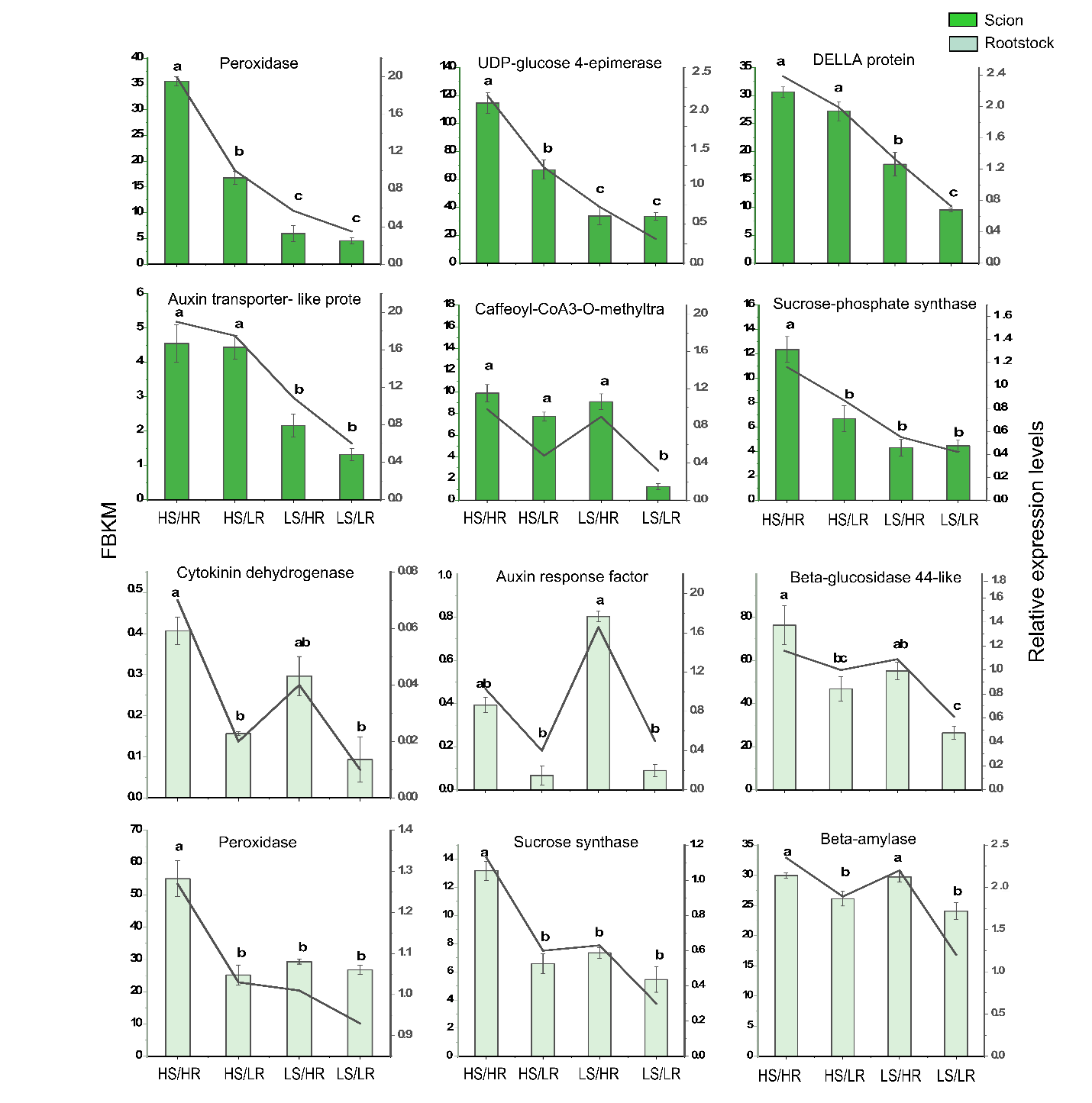


**Fig. S9.** Validation of RNA-sequencing data via quantitative reverse transcription polymerase chain reaction (qRT-PCR). Expression profiles of differentially expressed genes (DEGs) related to hormone signaling, phenylpropanoid biosynthesis, and carbohydrate metabolism pathways were validated using qRT-PCR in grafted watermelon under different light intensity. The y-axis shows qRT-PCR results (black line) alongside RNA-seq FPKM values (histogram), while the x-axis denotes samples.





**Fig. S10.** Heatmap of hub genes from the MEturquoise module associated with light-regulated graft healing in scion and rootstock tissues in response to light intensity. Hub genes identified from the MEturquoise co-expression module (via WGCNA) are shown for both scion (left) and rootstock (right) tissues. Expression levels are visualized as Z-score normalized values, with red indicating upregulation and blue indicating downregulation. Genes include key regulators of the cell cycle, hormone signaling pathways (auxin and cytokinin), enzymes related to carbohydrate metabolism and enzymes involved in phenylpropanoid biosynthesis, particularly those associated with lignin formation, highlighting their potential roles in light-mediated graft healing processes.





**Fig. S11.** Integrated analysis of differentially expressed genes (DEGs) and differentially accumulated metabolites (DAMs) in the phenylpropanoid biosynthesis pathway for watermelon and pumpkin. The rectangular boxes represent differential gene expression levels across the HS/HR, HS/LR, LS/HR, and LS/LR treatments, displayed sequentially from left to right. The heatmap visualizes FPKM values for DEGs, with red indicating upregulation and blue indicating downregulation.





**Fig. S12.** Integrated analysis of differentially expressed genes (DEGs) and differentially accumulated metabolites (DAMs) in the starch and sucrose metabolism pathway for watermelon and pumpkin. The rectangular boxes represent differential gene expression levels across the HS/HR, HS/LR, LS/HR, and LS/LR treatments, displayed sequentially from left to right. The heatmap visualizes FPKM values for DEGs, with red indicating upregulation and blue indicating downregulation.


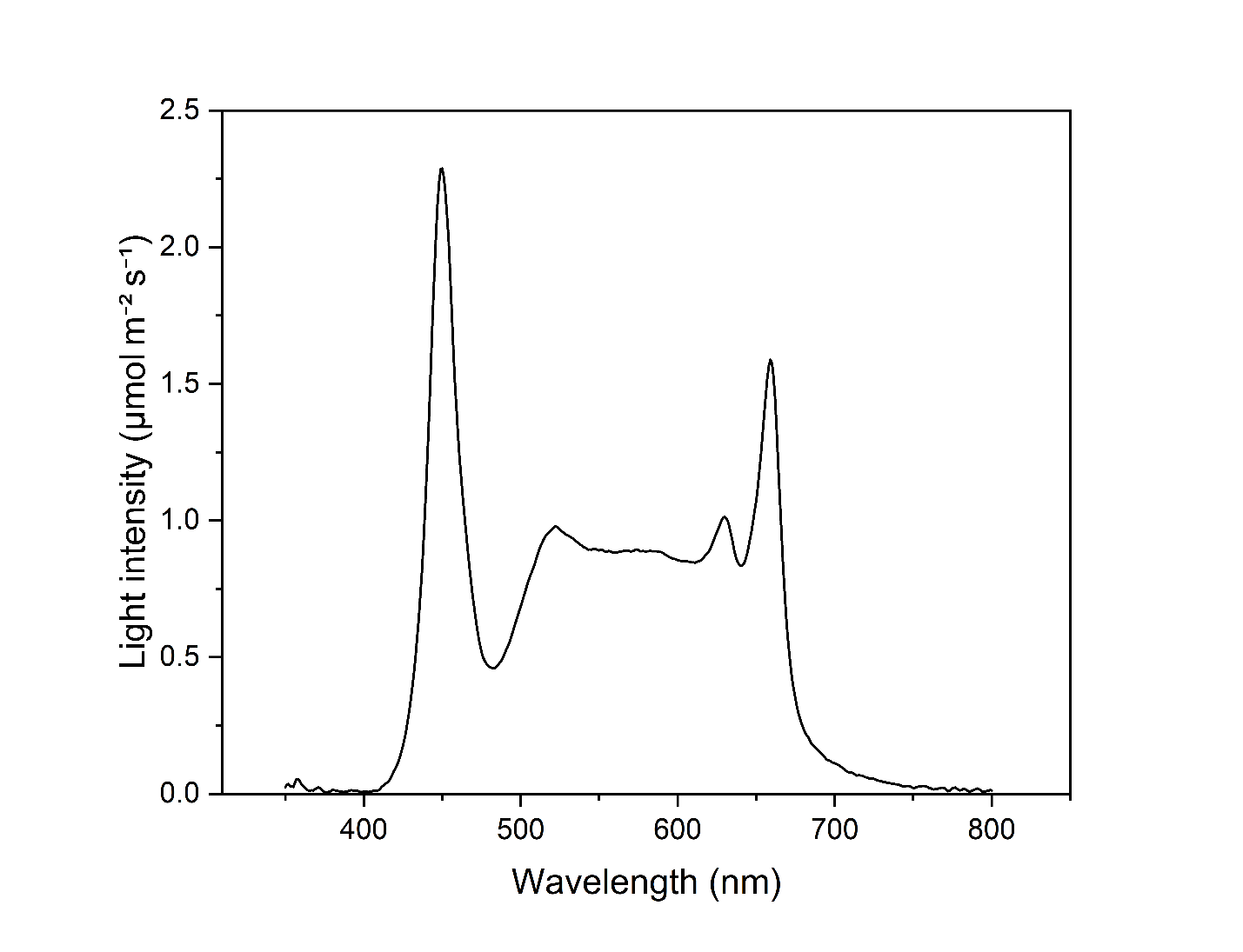


**Fig. S13**. Spectral distribution of the white LED light used in the plant factory with artificial lighting (PFAL). The spectrum shows light intensity (µmol m⁻² s⁻¹) across wavelengths ranging from 350 to 800 nm. Distinct peaks are observed in the blue (~450 nm) and red (~660 nm) regions.


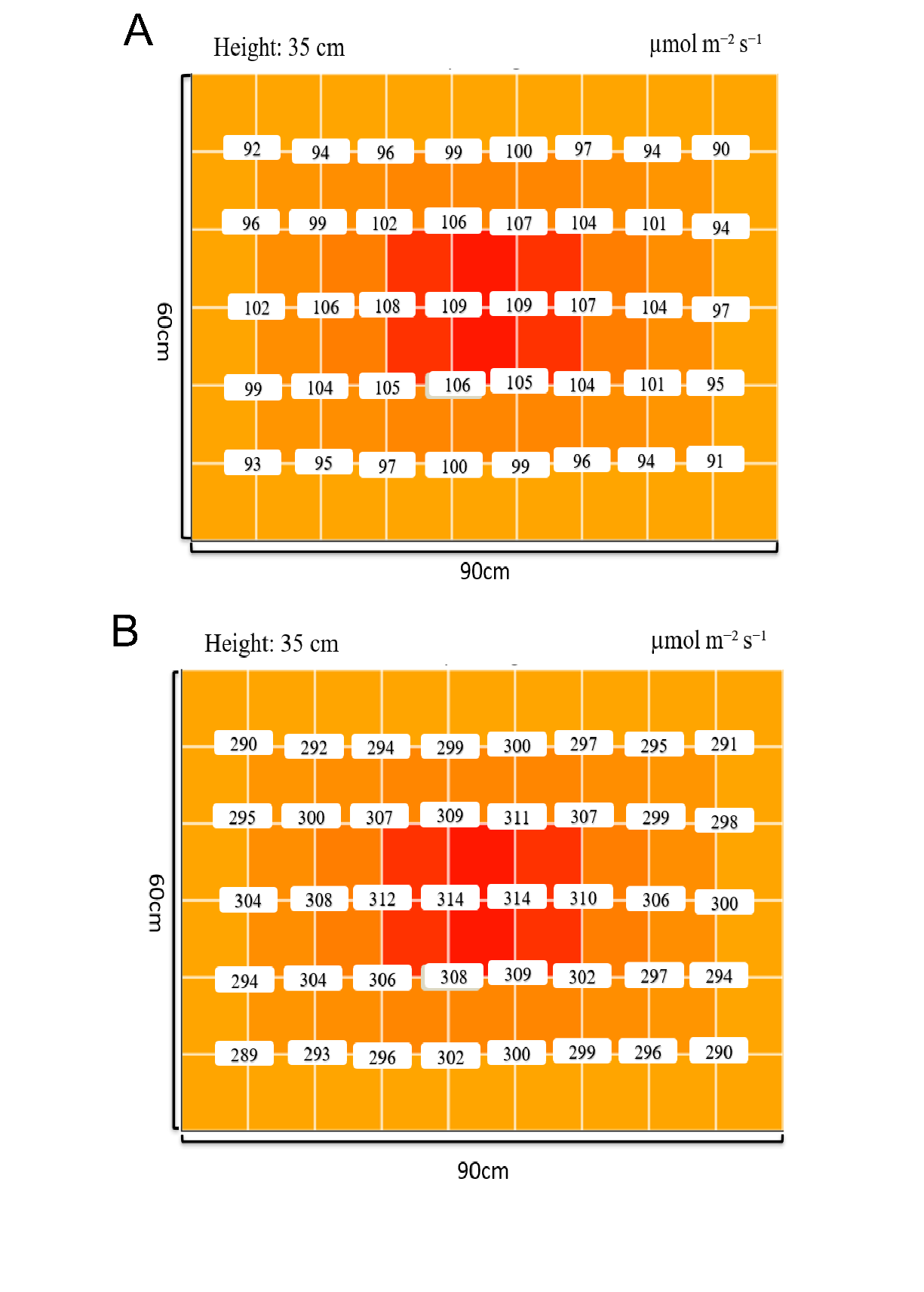


**Fig. S14.** Photosynthetic photon flux density (PPFD) distribution map at 35cm height from the plant top and over a 60 cm × 90 cm growth area. A, Low light intensity condition (~100 µmol m⁻² s⁻¹).
B, High light intensity condition (~300 µmol m⁻² s⁻¹). Color shading indicates gradient levels, with red representing higher intensity regions and yellow/orange indicating lower intensity zones.
